# Development of a novel streamlined workflow (AACRE) and database (inCREDBle) for genomic analysis of carbapenem-resistant Enterobacterales

**DOI:** 10.1099/mgen.0.001132

**Published:** 2023-11-27

**Authors:** Tyler S. Alioto, Marta Gut, Bruno Kotska Rodiño-Janeiro, Fernando Cruz, Jèssica Gómez-Garrido, Juan Carlos Vázquez-Ucha, Caterina Mata, Regina Antoni, Ferran Briansó, Marc Dabad, Eloi Casals, Matthew Ingham, Miguel Álvarez-Tejado, Germán Bou, Ivo G. Gut

**Affiliations:** ^1^​ Centro Nacional de Análisis Genómico, C/Baldiri Reixac 4, 08028 Barcelona, Spain; ^2^​ Universitat de Barcelona (UB), Barcelona, Spain; ^3^​ Microbiology Department, Complejo Hospitalario Universitario A Coruña-Instituto Investigación Biomédica A Coruña (INIBIC), A Coruña, Spain; ^4^​ CIBER de Enfermedades Infecciosas (CIBERINFEC), ISCIII, Madrid, Spain; ^5^​ Department of Genetics, Microbiology and Statistics, Universitat de Barcelona (UB), Barcelona, Spain; ^6^​ Roche Diagnostics, Sant Cugat del Vallès, Barcelona, Spain

**Keywords:** antimicrobial resistance, carbapenem-resistant Enterobacterales, database, genome assembly, nanopore sequencing

## Abstract

In response to the threat of increasing antimicrobial resistance, we must increase the amount of available high-quality genomic data gathered on antibiotic-resistant bacteria. To this end, we developed an integrated pipeline for high-throughput long-read sequencing, assembly, annotation and analysis of bacterial isolates and used it to generate a large genomic data set of carbapenemase-producing Enterobacterales (CPE) isolates collected in Spain. The set of 461 isolates were sequenced with a combination of both Illumina and Oxford Nanopore Technologies (ONT) DNA sequencing technologies in order to provide genomic context for chromosomal loci and, most importantly, structural resolution of plasmids, important determinants for transmission of antimicrobial resistance. We developed an informatics pipeline called Assembly and Annotation of Carbapenem-Resistant Enterobacteriaceae (AACRE) for the full assembly and annotation of the bacterial genomes and their complement of plasmids. To explore the resulting genomic data set, we developed a new database called inCREDBle that not only stores the genomic data, but provides unique ways to filter and compare data, enabling comparative genomic analyses at the level of chromosomes, plasmids and individual genes. We identified a new sequence type, ST5000, and discovered a genomic locus unique to ST15 that may be linked to its increased spread in the population. In addition to our major objective of generating a large regional data set, we took the opportunity to compare the effects of sample quality and sequencing methods, including R9 versus R10 nanopore chemistry, on genome assembly and annotation quality. We conclude that converting short-read and hybrid microbial sequencing and assembly workflows to the latest nanopore chemistry will further reduce processing time and cost, truly enabling the routine monitoring of resistance transmission patterns at the resolution of complete chromosomes and plasmids.

## Significance as a BioResource to the community

We provide the complete, gap-free and annotated sequences of 461 antibiotic-resistant bacterial strains collected from hospitals throughout Spain using the latest sequencing technology and assembly methods. We have developed a new database that tightly associates the clinical, geographical, microbiological and genomic data, and complements other online resources focused on antimicrobial resistance (AMR). In this paper we present the data set as a genomic resource, including a detailed characterization of the entire set of genomes of the carbapenem-resistant bacterial strains. Moreover, we communicate the laboratory, informatic and bioinformatic developments [the informatics pipeline Assembly and Annotation of Carbapenem-Resistant Enterobacteriaceae (AACRE) coupled with the inCREDBle database] that constitute a new tool for investigating AMR and represent a significant step toward routine genomic monitoring of antimicrobial resistance.

## Data Summary

The raw data (Illumina and ONT sequence reads) and annotated assemblies corresponding to 461 carbapenem-resistant Enterobacterales (CRE) genomes sequenced in this study have been submitted to the European Nucleotide Archive under BioProject accessions PRJEB42440 and PRJEB39112, with the latter project containing the data for 24 of the samples that have RNA methyltransferases (RMTases) in their genomes. The genomic data, along with the associated clinical and phenotypic data, are available for querying, browsing and downloading in a specialized CRE database portal called inCREDBle, available at https://genomes.cnag.cat/incredble/. In addition, for each sample, the following files are made available for download:

- <sample>.assembly.fasta.gz
- <sample>.annotation.gff3.gz
- <sample>.protein.fasta.gz
- <sample>.CDS.fasta.gz

Code developed:

- Database and analysis code: https://github.com/cnag-aat/incredble
- Assembly and annotation pipeline: https://github.com/cnag-aat/AACRE

Additional comparisons were made to the external set of *Klebsiella pneumoniae* genome assemblies corresponding to BioProject accession PRJEB10018. The authors confirm all supporting data, code and protocols have been provided within the article or through supplementary data files.

## Introduction

Infections with antibiotic-resistant bacteria represent a health concern that threatens worldwide public health, prompting the development of a coordinated action plan from the World Health Organization (WHO) to address it [1]. This multi-pronged action plan sets out several objectives, the second of which is to ‘strengthen the knowledge and evidence base through surveillance and research’ [1]. In addition, the WHO has specifically rated carbapenem-resistant *Acinetobacter baumannii *, *Pseudomonas aeruginosa* and Enterobacterales (which include *Klebsiella pneumoniae* and *Escherichia coli *) as pathogens of critical priority for the development of new antibiotics [2]. For serious Gram-negative bacterial infections, carbapenems are often considered the therapeutic agent of last resort, thus resistance to this class of drugs is highly concerning [3]. The clinical burden of antibiotic-resistant bacteria has increased from 2007 through 2015; in particular, the proportion of infections by carbapenem-resistant bacteria in Europe increased from 18 % in 2007 to 28 % in 2015 [4]. Furthermore, the increase in deaths was led by carbapenem-resistant *K. pneumoniae* (a 6.16-fold increase in deaths from 2007 to 2015) and *E. coli* (a 4.76-fold increase in deaths from 2007 to 2015), highlighting the relevance of these pathogens [4]. Thus, it is critically important that we discover and study in more depth the mechanisms by which antimicrobial resistance is being acquired or developed by species of the order Enterobacterales.

Systematic genome sequencing is an effective way to increase surveillance as well as to understand the successful expansion of the antibiotic-resistant bacteria associated with high-risk clones. Recent developments in sequencing technology have enabled the genomic analysis of relevant pathogens such as those of the order Enterobacterales. Recently, an epidemiological study of the distribution of >1700 *K *. *pneumoniae* strains isolated from patients from 32 European countries (EuSCAPE interim data set) demonstrated the role of carbapenemases (β-lactamase enzymes that hydrolyze carbapenems) in their widespread dissemination [5]. The main factor contributing to carbapenem resistance in Enterobacterales is the presence of carbapenemase enzymes, while secondary factors include efflux pumps, mutations in porins, mutations in penicillin-binding proteins (PBPs) and the presence of other β-lactamases [6]. Carbapenemases have the ability to spread quickly due to their association with mobile elements (e.g. transposons, integrons, etc.) and their presence in conjugative or mobilizable plasmids, which are the principal factors responsible for horizontal transmission of antimicrobial resistance [7].

However, while substantial progress has been made in the sequencing of carbapenem-resistant Enterobacterales (CRE), their flexible or accessory genomic regions, including plasmids, are in general poorly characterized. The use of short-read sequencing results in fragmented assemblies that do not reach the necessary contiguity and completeness to accurately reconstruct these regions (e.g. plasmids or structural variants) [8]. This shortcoming is non-trivial in the case of Enterobacterales, which present high genomic plasticity [9]. Better assembly of the flexible genome has the potential to yield more precise information about antimicrobial resistance, virulence factors and differential characteristics among strains, illuminating the genetic underpinnings of high-risk clones [10]. Many of the limitations of short-read assemblies are addressed using hybrid assembly methods, which combine the advantages of short reads (low error rate) and long reads (resolution of complex repeats) [11, 12]. Long-read assemblies allow the precise definition of the genomic elements containing mobile carbapenem-resistance systems and/or virulence factors and also enable more detailed characterization of different Enterobacterales sequence types. The complete and accurate assembly of carbapenemase-encoding plasmids is uniquely achieved by long-read or hybrid assembly [11].

For genomic data to be useful for surveillance or for in-depth analysis requiring large data sets, its availability in well-structured relational databases is an equally important requirement. As mentioned above, the currently available data on antimicrobial resistance (AMR) are highly skewed toward short-read assembly data. To address these shortcomings with respect to high-quality AMR genomic data in Spain, we aimed to produce complete genome assemblies utilizing both long-read and short-read sequencing for over 450 CPE isolates from Spain and develop a database that would facilitate the analysis of resistance and virulence mechanisms. A secondary objective was to optimize the sequencing and assembly strategy, varying length, depth and quality, for the production of high-quality bacterial genome assemblies.

Here we describe the result of this effort: a set of genomic, clinical and phenotypic data for 461 strains isolated from 41 hospitals across 13 different regions of Spain, deposited in the International Nucleotide Sequence Database Collaboration (INSDC) sequence repositories, and made available for interactive exploration via a specialized CRE database portal called inCREDBle, available at https://genomes.cnag.cat/incredble/. The isolates were fully sequenced with a combination of long Oxford Nanopore Technologies (ONT) and short Illumina reads. The data set represents a snapshot of the distribution, characteristics and mechanisms of resistance to carbapenem antibiotics in Enterobacterales in Spain. The streamlined workflow and database described here have been developed so that, if integrated directly with clinical microbiology services and updated routinely, they will have the ability to greatly improve the quality of AMR surveillance with real-time efficiency.

## Methods

### CRE genomic database (inCREDBle)

To store and be able to analyse the assembly and annotation results along with the associated clinical and phenotypic information, we designed a database (Fig. S1, available in the online version of this article) and web-accessible front-end called inCREDBle (https://genomes.cnag.cat/incredble/) using Django, a high-level Python-based web application framework. The source code for the web application is available at https://github.com/cnag-aat/incredble. The MySQL database can be queried directly with SQL or accessed via a REST API, but the most power and flexibility are afforded by the Django Python framework. Via the Django shell or Python scripts, flexible queries can be constructed. For the end user, though, we developed a flexible user-friendly web front end for mining the database, allowing searches and filtering on samples (clinical data, species, sequence type, etc.), sequences (blast/MASH), sequence classification (length, circularity, replicons, mob type, origin, etc.), or gene annotations. An additional emphasis has been placed on comparative genomic features: navigation among similar sequences (plasmids, chromosomes) is facilitated by the incorporation of MASH [12] clusters, and navigation among homologous genes is enabled by the incorporation of Roary [13] clusters. In addition to tabular data, we include visualizations in the form of charts, maps and interactive networks. Finally, for better understanding of genomic context, the database is linked to a genome browser.

### Collection, culture and DNA extraction

In 2018 we compiled a representative set of 499 clinical isolates of carbapenemase-producing Enterobacterales (CPE) recovered from patients with proven infection/colonization from 41 participating hospitals from 13 different Spanish regions that had been isolated over a period of 6 years. The majority were collected over a period of 2 months in 2017 from 24 of the over 400 hospitals in the Spanish National Health System. The selected hospitals were mostly third-level hospitals, highly specialized, and with more than 300 beds. The main criteria for inclusion were meropenem minimum inhibitory concentration (MIC) greater than 0.125 mg l^−1^ (screening cut-off value for CPE) and the detection of a carbapenemase by phenotypic or molecular methods, following the European Committee on Antimicrobial Susceptibility Testing (EUCAST) recommendations. The detection of carbapenemase enzymes was performed by PCR and phenotypic methods before sequencing. The remainder of the isolates included in this study correspond to KPC-positive isolates collected by the Spanish Centro Nacional de Microbiología (CNM) from 2012 to 2017 and with the same inclusion criteria.

The Complexo Hospitalario Universitario de A Coruña (A Coruña, Spain) served as a reference laboratory. Only non-identifying data such as geographical origin, sample type and sex, were recorded for each isolate and deposited in inCREDBle. Bacterial strains were received and frozen in Luria–Bertani (LB) broth with 10 % glycerol. Strains were maintained at 80 °C until analysis.

The Enterobacterales strains were plated on LB broth agar overnight at 37 °C. Two 5 ml LB broth tubes were inoculated with one colony each and incubated in a shaker at 37 °C overnight. The duplicate overnight cultures were spun down at 3000 relative centrifugal force (RCF) for 10 min and the supernatant was discarded. DNA was extracted from both pellets with the Genomic DNA Buffer Set and Genomic-tip 20/G (Qiagen) using lysozyme (140 000 units, Sigma-Aldrich) following the manufacturer’s instructions. The quantity of DNA was measured with the HS dsDNA Assay kit by Qubit (Thermo Fisher Scientific) and its integrity was assayed by 0.5 % agarose gel electrophoresis.

### Short-read whole-genome sequencing

The short-insert paired-end libraries for the whole-genome sequencing were prepared with a PCR-free protocol using the KAPA HyperPrep kit (Roche) with some modifications. As a function of the amount of available material, 0.2–1.0 µg of genomic DNA was sheared on a Covaris LE220-Plus (Covaris), size-selected and xGen UDI-UMI Adapters (Integrated DNA Technologies) were ligated. The libraries were quality controlled on an Agilent 2100 Bioanalyzer with the DNA 7500 assay (Agilent) for size and quantified using the Kapa Library Quantification kit for Illumina platforms (Roche).

The libraries were sequenced on NovaSeq 6000 (Illumina) in paired-end mode following the manufacturer’s protocol for dual indexing. Image analysis, base-calling and quality scoring of the run were performed using the manufacturer’s software Real Time Analysis (RTA 3.4.4) and FASTQ sequence files were generated.

### Long-read whole-genome sequencing

Genomic DNA from CRE was used to prepare 1D native barcoding genomic libraries using the ligation sequencing kit SQK-LSK109 plus the native barcoding expansion kits EXP-NBD103 (1–12), EXP-NBD104 (1–12) and EXP-NBD114 (13–24) from Oxford Nanopore Technologies (ONT).

Samples were processed following the manufacturer’s recommendations for R9.4.1 (R9) flow cells. Library preparation was performed with 1.5 µg of genomic DNA without previous sample fragmentation. DNA repair and end-prep of the samples were performed in the same reaction using the NEBNext FFPE DNA Repair Mix (NEB, M6630) and the NEBNext UltraII End Repair/dA-Tailing Module (NEB, E7546), followed by a 1× AMPure XP Beads purification step.

Native barcodes were ligated 1 h at room temperature (RT) to the purified DNA using the NEB Blunt/TA ligase Master Mix (NEB, M0367L) and purified with 1× AMPure XP Beads. Once purified, a 1 µg equimolar pool of the barcoded samples was prepared to follow up with the ligation of the sequencing adapter carrying the motor protein. The adapter ligation step was performed using the NEBNext Quick Ligation Module (NEB, E6056) and the sequencing adapter (AMX II or BAM 1D according to the barcoding kit used) at RT for 1 h. The ligation of the adapters was followed by a 0.4× AMPure XP Beads final purification step, washed twice with Short Fragment Buffer (SFB) (SQK-LSK109) and eluted in 14 µl of elution buffer (SQK-LSK109).

Seventy-six samples were selected and prepared for sequencing again on R10.3 (R10) flow cells. This data set included 56 samples that were sub-optimally assembled as well as 20 samples with fully circularized assemblies to serve as positive controls. Representation of different species served as additional criteria for selection of this set of samples (Table S5). The library preparation protocol only differed from the R9 protocol in the amount of genomic DNA used as starting material, which was 2.5 µg, and the amount of the equimolar pool of barcoded samples used for adapter ligation, which was 2.5 µg.

Quality control of the resulting libraries was performed by analysing a 1 µl aliquot of adapter ligated DNA by Qubit fluorometry and Agilent 2100 Bioanalyzer.

For the sequencing runs performed on R9 FLO-MIN106 or FLO-MIN106D flow cells, 600 ng of the final library containing an average of 24 pooled samples was loaded. For the sequencing runs performed on R10 FLO-MIN111 flow cells, the amount of pooled library loaded containing an average of 12 samples was 350 ng. After priming the flow cell, the pre-sequencing mix was combined with 37.5 µl of Sequencing Running Buffer (SQK-LSK109) and 25.5 µl of Library Loading Beads (SQK-LSK109).

The different flow cells used were connected either on a MinION/MK1B or in a GridION X5 instrument (ONT). For all libraries, the MinKNOW interface QC (ONT) was run in order to assess the flow cell quality. Once the priming of the flow cell was finished, the libraries were loaded into the flow cells and the sequencing data were collected after 48–90 h. The quality parameters of the sequencing runs were further monitored by the MinKNOW platform in real time. For the runs launched on the MinION/MK1B, the MinKNOW versions used were 1.15.4 and 3.1.19 and the base calling was performed after the end of the run using Guppy versions 2.3.5 and 2.3.7. For the runs launched on the GridION X5, the MinKNOW versions used were 3.3.2 and 3.5.4 and the base calling was done during the run using the Guppy versions 3.0.3 and 3.2.6. All runs performed with R10 flow cells were launched on the GridION X5 instrument using MinKNOW version 3.6.5. The real-time base-calling Guppy version was 3.2.10.

### Assembly and annotation pipeline

A Snakemake workflow [14], which we have named Assembly and Annotation of Carbapenem-Resistant Enterobacteriaceae (AACRE), was developed to automatically process, assemble and annotate the sequenced data. The source code for the pipeline is available at https://github.com/cnag-aat/AACRE. The steps are detailed further below.

For Illumina data, removal of adaptors and quality trimming was performed with Trim Galore! [15] v0.3.3 (using -q 10 and --length 100), while ONT barcode removal was performed with qcat [16] v1.0.1 (specifying --detect-middle and --trim options). Contaminants present in the raw reads were identified using Kraken [17] v2.0.7 and their abundance at the species level was computed with Bracken [18] v2.0. In addition, the sequencing statistics of the de-barcoded ONT reads (used as input) were estimated with NanoPlot [19] v1.8.0 for each sample.

### Hybrid assembly

Illumina and ONT sequences were assembled with Unicycler [20] v0.4.6, a hybrid assembly pipeline designed for microbial genome assembly that leverages the advantages of both long and short reads and outputs circularized contigs (when possible) representing genomic chromosomes and associated plasmids. Unicycler was run in both normal and bold modes. In most cases, the normal-mode assemblies were selected for further analysis and inclusion in the inCREDBle database. However, in some cases the bold-mode assembly was clearly more contiguous and circularized, and was selected instead.

### Long-read assembly

A selected subset of 74 samples was sequenced again with ONT R10.3 pore chemistry. All data were downsampled to 100× coverage and reads shorter than 1 kb were filtered out. Long read-only assemblies were performed for a subset of 33 samples for which both R9 and R10 read data were available at sufficient coverage. Assemblies of R9 or R10 reads or combinations thereof were performed with Flye [21] v2.6 with the parameters -i 2 --plasmids --meta --nano-raw <reads>. The resulting assemblies were polished with two rounds of Racon [22] v1.3.1 and one round of Medaka [23] v1.0.3 using the appropriate pore- and guppy-specific model: r103_min_high_g345 or r941_min_high_g344 (in the case of mixed R9/R10 reads, only the R10 reads and corresponding R10 model were used). Further polishing with 100× of downsampled Illumina reads was performed with Pilon [24] v1.21. Accuracy was measured by aligning the resulting assemblies to the hybrid assembly (previously obtained using all generated ONT R9 and Illumina data) using dnadiff from the Mummer package [25].

### Annotation

The assembled genomes were annotated using PROKKA [26] v1.12, specifying the kingdom, genus and species in addition to default options. In addition, several tools were run to obtain a comprehensive annotation of the genes involved in conferring antibiotic resistance. Resistance Gene Identifier (RGI) [27], with CARD database version 3.0.2, was used to predict resistomes from the genome assembly. The Prokka and RGI annotations were combined to produce a final protein-coding gene set. Furthermore, AMRFinderPlus [28] v3.6.10 was run on the annotated proteins to include the annotation of antimicrobial-resistant genes from the National Center for Biotechnology Information (NCBI) database. Resfinder [29] v3.0 was also run on each assembly.

To annotate insertion sequences (ISs), we performed a blast search of each assembly against the Insertion Sequence database [30]. Centrifuge [31] v1.0.4-beta was used to search the assembled scaffolds (i.e. main chromosomes and plasmids) against the bacterial *nt* blast database [32]. Scanning of assemblies against traditional PubMLST typing schemes was carried out with the mlst program [33].

PlasmidFinder [34] and MOB-typer from MOB-Suite v2.1.0 [35] were run to identify relaxase types and replicons. For scaffolds larger than 1 Mb identified as plasmid sequence (either putative insertions into bacterial chromosomes or misassemblies), we mapped all ONT reads as well as the plasmids belonging to four identified Mash clusters (429, 708, 770 and 3040) back to these 11 assemblies using minimap2 (version 2.14). The alignments were sorted and indexed with samtools v2.19 and then visualized with the Integrative Genomics Viewer (IGV).

For each plasmid with a disrupted gene flanking the contig start/end (site of linearization by Unicycler), we used progressiveMauve [36] v2.4.0 to align it to all other similar plasmids belonging to the same MOB-typer cluster (same MASH cluster, mob-type and replicon) and then used an in-house script to select an appropriate start position.

### Multilocus sequence typing

Multilocus sequence types (MLSTs) were determined *in silico* [33] from assembled whole-genome sequencing data using available PubMLST databases. New MLST combinations were submitted to the respective PubMLST databases: *Citrobacter freundii *, *Enterobacter cloacae* complex and *Klebsiella oxytoca* were submitted to PubMLST [37] and *K. pneumoniae* was submitted to Institut Pasteur [38].

### Species identification

The initial characterization of species with matrix-assisted laser desorption/ionization time-of-flight mass spectrometry (MALDI-TOF MS) or in some cases by biochemical tests or by analytical profile index (API) was cross-validated with the sequencing results. The results of SpeciesFinder, multilocus sequence typing-identified species and the results from MALDI-TOF MS were used to check contamination and to assign the final taxonomic classification. In the cases of closely related species, a curation based on core genome phylogeny was applied.

### Identification of carbapenamase genes

Following EUCAST recommendations (https://www.eucast.org/resistance_mechanisms/), the detection of carbapenemases in the hospitals of origin was performed by PCR with specific primers for the most prevalent carbapenemase genes (KPC, OXA-48, NDM, VIM, IMP) [39] and/or by phenotypic methods such as the modified Hodge test, and biochemical (colorimetric) tests such as CARBA-NP.

After sequencing and assembly, we combined the output of AMRFinder, RGI and ResFinder to verify the presence and correct annotation of the carbapenem resistance genes previously predicted to be present by the hospitals of origin: phenotypic tests (AST, EDTA) and molecular tests (MALDI-TOF, PCR), referred to subsequently as the ‘metadata’. Discrepant cases, where the annotation of the carbapenemase produced by the gene prediction programs (GPs) did not match the metadata, were selected for confirmation by PCR. We excluded samples from the data set if: (1) the carbapenemase could not be confirmed with either annotation or PCR or (2) the carbapenemase(s) detected by PCR were not the same as the annotated one(s). Samples in which additional carbapenemases were found with respect to the metadata or PCR were kept. Of the 499 clinical isolates collected, 38 were removed. In addition, we identified 16 plasmid sequences with only partial carbapenemases detected near the beginning or end of the FASTA sequences; to solve this specific annotation problem, we moved the origins of these plasmids and the rotated FASTAs were re-annotated, resulting in corrected carbapenemase gene annotations.

### Phylogenetic inference

Core and accessory genomes of the three most abundant species (64 *E. cloacae complex*, 24 *E. coli* and 332 *K *. *pneumoniae* isolates) were obtained with Roary [13] v3.13.0. The core genome alignments generated by Roary included a total of 1957 genes from *E. cloacae *, 3119 genes from *E. coli* and 3831 genes from *K. pneumoniae *. The phylogenetic tree of each alignment was generated with RaxML [40] v8.2.12 using a general time-reversible model with a gamma correction for site rate variation and the options: -f a (for rapid bootstrap analysis), -p 12 345 (random seed for parsimony inference), -x 12 345 (random seed for bootstrapping) and -# autoMRE to adapt the number of bootstrap iterations to obtain the convergent tree. The phylogenetic tree was visualized together with associated metadata using iTOL [41] v5.

Enrichment analysis of genes specific to or absent from genomes of a particular sequence type was performed as follows. For each Roary group identified, a two-by-two contingency table was constructed with the number of distinct samples that were positive or negative for the gene in the sequence type of interest versus the number of samples that were positive or negative for the rest of the sequence types of the same species. *P*-values were obtained with Fisher’s exact test. As we were interested in the most extreme differences in distribution, we additionally applied a threshold of 85 % positive (present) in the test group (ST15, for example) and <5 % positive (present) in the rest of the samples. Code is available at https://github.com/cnag-aat/incredble/blob/master/scripts/enrichment.py.

Plasmids, the main contributor to the accessory genome, were clustered using pairwise average nucleotide identity (ANI) [42] and alignment fraction (AF). The ANI measure was first conceived for analysing bacterial core genomes, but the ANI and AF values can be combined to cluster plasmids. For this purpose, we followed the approach previously described [43] and downloaded the necessary scripts from https://github.com/santirdnd/ptu_paper/. Briefly, to obtain the AF value, pairwise blast searches were performed, with each sequence divided into 250 bp fragments with a 50 bp step. Similar blast searches were performed for determining the ANI values, but in this case 1 kb fragments from each sequence (with a 200 bp step) were used. After obtaining the AF and ANI global values for each pairwise combination, the total ANI [43 ], defined as −ln(AF×ANI) , was calculated and clusters of highly similar plasmids (tANI <0.5) were obtained. Results were visualized with the networkxx and plotly Python modules.

Orthofinder [44] was run to determine orthologous relationships between all the genes in the data set. Orthogroups, groups of genes that are orthologous, were added to the inCREDBle database to allow the search of all the orthologous genes of a gene of interest.

### Comparison with short-read assemblies

To examine the advantages and disadvantages of the hybrid versus Illumina-only strategies for assembling bacteria, we first compared the final Unicycler hybrid assemblies to the intermediate Illumina-only assemblies produced by SPAdes within the Unicycler pipeline using the same data set of 332 *K *. *pneumoniae* samples, allowing us to see the direct benefits of ONT data incorporation. For this purpose, we ran the same annotation steps that were previously run in the hybrid assembly. In addition, we compared our 332 *K *. *pneumoniae* hybrid assemblies to a previously published data set of 1717 carbapenem-producing *K. pneumoniae* samples sequenced and assembled with an Illumina-only strategy [5 ]. We ran the 1717 Illumina-only assemblies (PRJEB10018/ERP011196) through the same annotation pipeline used for our hybrid assemblies, and then we compared the gene and IS annotations with those of the 329 *K *. *pneumoniae* genome sequences in our data set.

## Results

### Sample characteristics

**Fig. 1.**
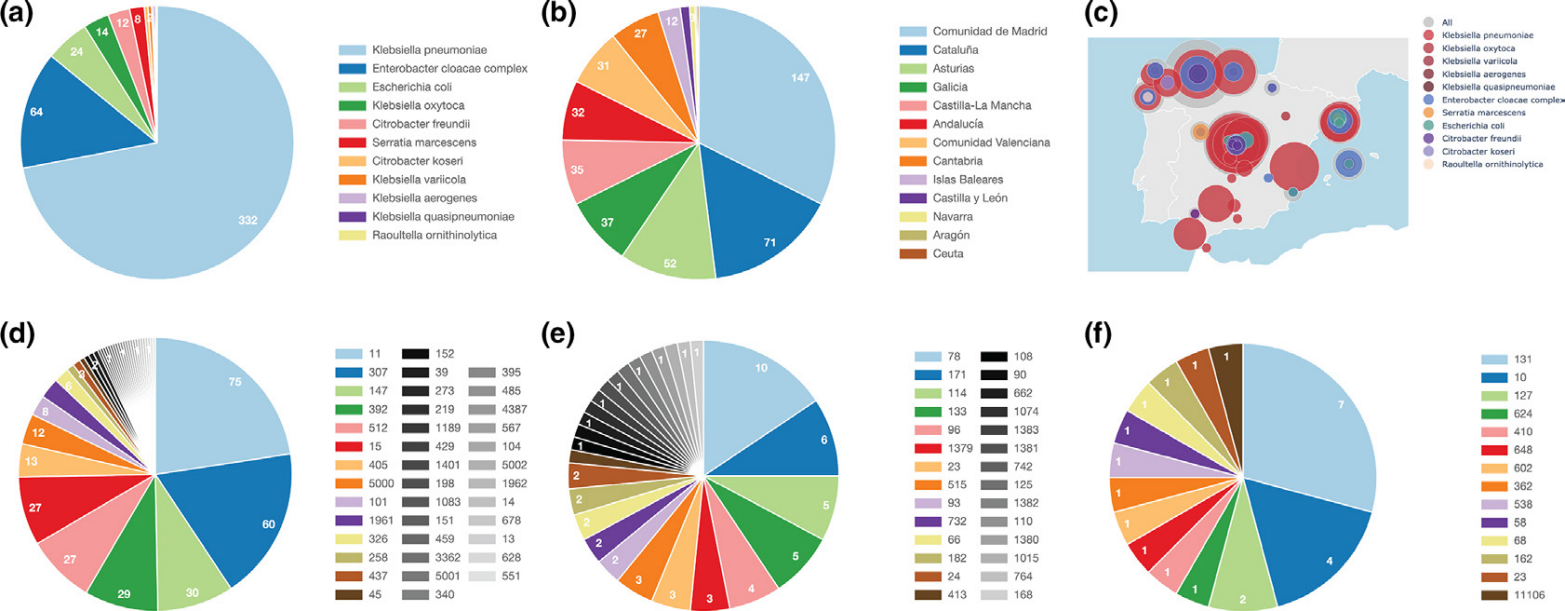
Distribution of (**a**) species, (**b**) communities and (**c**) species by geographical location on a map of Spain, and sequence types for (**d**) *K. pneumoniae *, (**e**) *E. cloacae* and (**f**) *E. coli *.

A total of 461 of the samples collected (92.4 %) were successfully sequenced with both Illumina and Oxford Nanopore Technologies platforms, assembled, annotated and included in the data set hosted at https://genomes.cnag.cat/incredble/. The isolates were obtained from 41 Spanish hospitals in 13 different Spanish regions (Fig. 1), with almost 75 % of the isolates obtained from only five regions: Comunidad de Madrid (31.9 %), Cataluña (15.4 %), Asturias (11.3 %), Castilla-La Mancha (7.6 %) and Galicia (7.8 %). Almost all isolates (97.6 %) were associated with infections, while the remaining 2.4 % were derived from colonizations, i.e. the presence of bacteria in or on the body surface not causing any signs or symptoms of disease. Four hundred and ten (89 %) were community-acquired, meaning that the bacteria were acquired outside of any point of care, 47 were hospital-acquired and 4 were acquired within long-term care facilities. The most frequent sample of isolation was urine (252 isolates, 54.7 %) followed by wound (65 isolates, 14.1 %) and blood culture (46 isolates, 10.0 %).

**Fig. 2.**
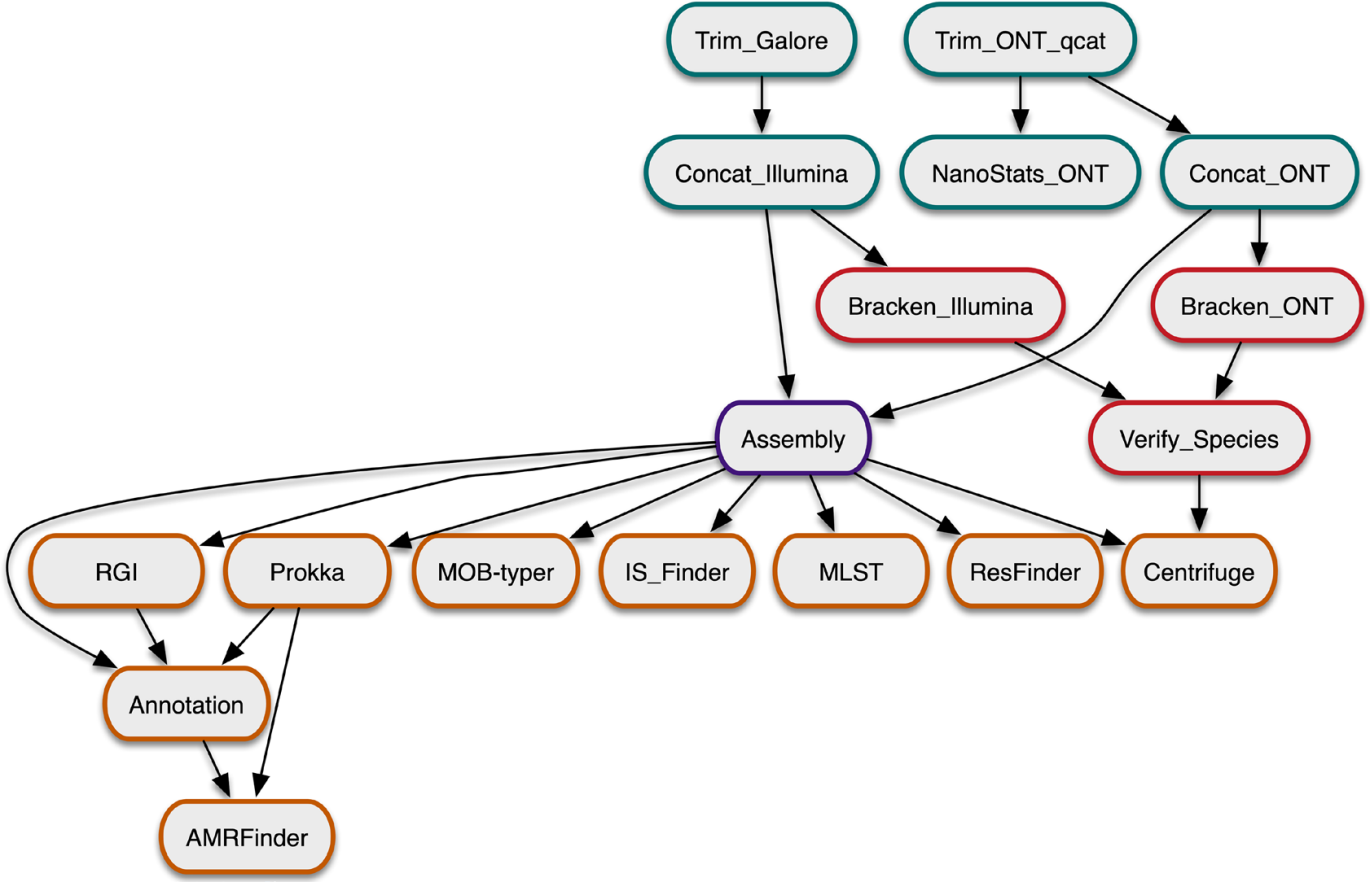
The AACRE workflow. Pre-processing steps are shown in green. Pre-assembly quality control steps to verify the purity and identity of the samples are shown in red. Assembly by Unicycler is shown in purple. Annotation steps are shown in orange.

To uniformly assemble and annotate these CRE isolate genomes, we developed the Assembly and Annotation of Carbapenem-Resistant Enterobacterales (AACRE) pipeline, which performs quality control, hybrid assembly with Unicycler followed by annotation of sequence type, genes, repeats and plasmids, with particular attention to antimicrobial resistance. A schematic of the AACRE workflow is shown in Fig. 2. The AACRE pipeline was run on all samples and the results stored in inCREDBle, a database we developed that combines the clinical, geographical, microbiological and genomic data of the CRE isolates we sequenced. The pipeline took a total of 11+/−5 h per sample (Fig. S2). This time was the result of adding each step’s running time, but as some of the steps were run in parallel, the total CPU time was higher (mean=48.5 h, sd=25 h). The most limiting step in the pipeline was the assembly, being both the longest and the one using the most memory. By default, it was run with four CPUs and a time limit of 14 h, but this had to be increased for some of the samples. The longest assembly took 24 h with four CPUs. Fourteen samples required more memory for the assembly step, so they were re-run with 16 CPUs, thus completing faster.

### Assembly quality

The samples were sequenced at high depth (mean≥150×) with both short and long reads, resulting in a median assembly length of 5.64 Mb with >83 % of the assemblies including only circular contigs (Table S1). The distributions of Illumina versus ONT coverage were not significantly different (two-sample Kolmogorov–Smirnov test: D=0.0889, *P*-value=0.052, *n*=461) (Fig. S3). As expected, the hybrid assemblies were significantly more contiguous (Wilcoxon rank sum test with continuity correction *P*<2.2e-16 comparted to short-read assemblies of the same samples or to the 1717 *K *. *pneumoniae* assemblies of PRJEB10018 [5] (Table S2). The hybrid assemblies show a bimodal distribution for the scaffold length, with a second peak with a high proportion of scaffolds of bacterial chromosome length, whereas both SPADes assembly data sets do not (Fig.S4). The better assembly also translated to higher quality resistance gene annotation. In the hybrid assemblies ~25 % of resistance genes were found in short plasmid-length contigs, while ~75 % were located in large contigs corresponding to the main chromosome. In the Illumina-based assemblies, most of the resistance genes were found in sequences shorter than 1 Mbp, making it difficult to distinguish chromosomal from plasmid genes. Comparing our own intermediate short-read assemblies to the final hybrid assemblies, we found that the total number of genes annotated in the Unicycler hybrid assemblies was higher than that of the SPAdes Illumina-only assemblies (Table S3). While there was little difference in the number of resistance genes annotated by the Resistance Gene Identifier (RGI) software [27] (*P*-value=0.041), the number of complete RGI genes was significantly higher (*P*-value=2.9e-11) in the hybrid assemblies, suggesting that the gene models were of better quality. Direct comparison of the two annotations for each sample confirmed this observation (Fig. S5).

### ISs

The improvement in annotation quality also extended to non-coding elements. In our final set of *K. pneumoniae* hybrid assemblies, a total of 1.09 % of assembled genomic sequence was annotated as IS, while only 0.26 % of assembled genomic sequence was annotated as IS elements in the PRJEB10018 *K. pneumoniae* data set. IS-mediated transposition of associated resistance genes to new locations in the same or different DNA molecules is likely a key factor in the spread of antibiotic resistance [45]. We examined whether the Illumina-only assemblies are able to capture IS elements flanking resistance genes. We found that only 1.32 RGI-annotated genes per genome had IS elements within 1 kb upstream or downstream of them, while in our hybrid assemblies this number increased to 8.17 genes per genome. We also compared our own intermediate short-read assemblies to the final hybrid assemblies. Comparing the percentage of sequence covered by IS elements for all our hybrid and Illumina-only assemblies confirmed that IS elements were under-predicted in Illumina-only assemblies (Table S3).

### Impact of read length, yield and accuracy

Down-sampling of ONT read data showed that the depth of sequencing was sufficient to assemble the genomes and achieve fully circular main chromosomes plus plasmids for the vast majority of genomes (Fig. S6). Circularity ratio is a metric we define as the proportion of circular scaffolds of each assembly. It reflects the completeness of assembly of the main bacterial chromosome and plasmids for each sample. We found a positive and highly significant correlation between ONT coverage and circularity ratio as well as proportion of fully circular assemblies (Spearman’s rank correlation: rho=0.282 and rho=0.9636364, respectively, *P*-values <2.2e-16). We obtained ≥80 % fully circularized assemblies with ONT coverage of just 30×, and ~90 % with 60–80× coverage.

During our project, a new pore chemistry was released by Oxford Nanopore, testing of which became a secondary aim of the project. For a selected subset of 74 samples for which a sufficient quantity of DNA sample remained, we sequenced them again with the R10.3 pore chemistry. Samples were selected in order to cover a range of assembly ‘difficulties’ using the contiguity and circularity of the hybrid assemblies of R9 ONT data and Illumina as a proxy for ease of assembly. For the subset of more-difficult-to-assemble samples selected for sequencing with both R9 and R10, we observed a small increase in the number of assemblies with incompletely circularized contigs when using R10 data (Fig. S7), although pairwise comparisons of circularity ratios applying the Wilcoxon rank tests were not significant. Analysis of 10–100× coverage slices of both R9 and R10 read data showed a positive and significant correlation between ONT coverage and circularity (Spearman’s rank correlation: rho=0.31, *P*-value=3.02e-10). The generally lower circularity ratio seen in R10 was driven primarily by the output of a higher number of shorter linear scaffolds and only a slight reduction in the number of circular scaffolds (Fig. S8).

For those samples with incompletely circularized assemblies, 77 samples assembled using R9 data (Fig. S9) and 56 samples resequenced with R10 (Fig. S10), extremely low ONT read length N50 contributed to lack of circularity, suggesting that neither yield nor chemistry, but rather re-extracting higher-molecularweight DNA to achieve a significant fraction of reads longer than the golden 7 kb threshold [46], would have been beneficial for re-assembling these problematic samples.

### R10 pore chemistry improves consensus quality

While the assembly quality of hybrid assemblies did not improve with R10, the assembly quality of long read-only assemblies did, with comparable results to the hybrid assemblies in terms of accuracy. In comparison to R9 assemblies, R10 assemblies displayed higher consensus accuracy (Fig. S11), with a concomitant decrease in fragmented genes (Fig. S12a–d) and fewer missing resistance genes (Fig. S12e, f). The mean percentage identity for R10-based assemblies, 99.993 % (QV42), was significantly higher (Wilcoxon signed rank exact test: *P*-value=2.33e-10) than that of R9-based assemblies, 99.825 % (QV28). Polishing with Illumina reads brought the QV values up to 42 and 45 for R9 and R10 assemblies, respectively. Using a mix of 50× R9 and 50× R10 followed by ONT and Illumina polishing also achieved QV45. The extra boost from QV42 to QV45, however, was not necessary for improving the annotation of AMR genes (see below), as assemblies of R10 data alone missed very few AMR genes with respect to Unicycler hybrid assemblies.

### Sequence types identified and their genetic relationships

**Fig. 3.**
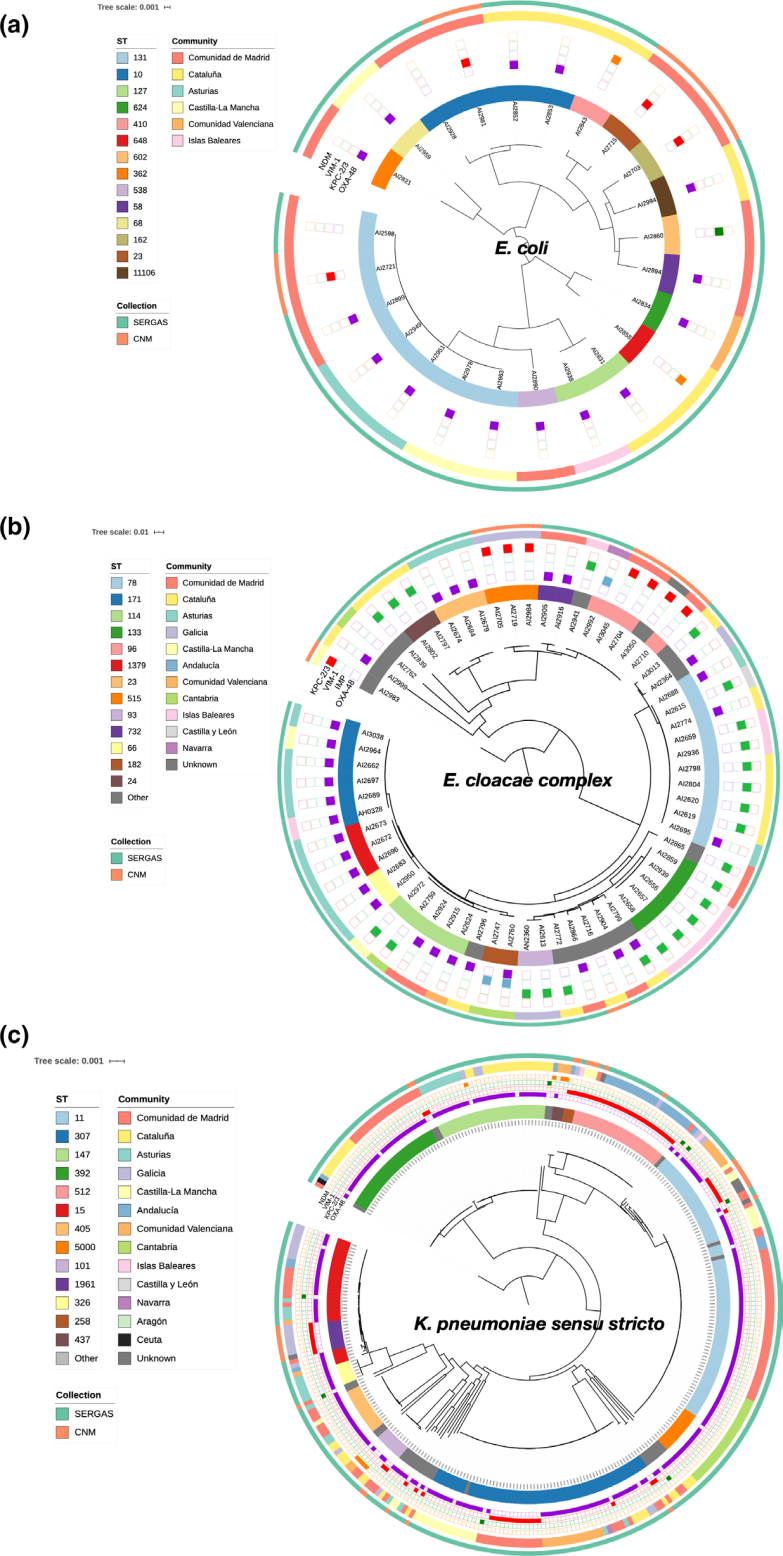
Phylogenetic relationship among isolates. Phylogenetic trees of core genomes for (**a**) *E. coli *, (**b**) *E. cloacae* complex and (**c**) *K. pneumoniae* were annotated with multilocus sequence type (ST), carbapenemases present (OXA-48, KPC-2/KPC-3, VIM-1, NDM or IMP), the community in Spain where strains were isolated and the collection from which they came (SERGAS or CNM). The *E. cloacae* complex includes the following species: *Enterobacter hormaechei* subsp *. hoffmannii* (ST78), *Enterobacter xiangfangensis* (ST171, ST114, ST1379, ST66, ST182), *Enterobacter roggenkampii* (ST96, ST515) and *Enterobacter hormaechei* subsp. *steigerwaltii* (ST93, ST133) [56, 57].

The most abundant species represented in the data set were *K. pneumoniae* sensu stricto (332, 72.0 %), *E. cloacae* complex (64, 13.9 %) and *E. coli* (24 isolates, 5.2 %). The most common MLSTs for *K. pneumoniae* were ST11 (22.6 %), ST307 (18.1 %) and ST258/512(9.0 %); for *E. cloacae* complex they were ST78 (* E. cloacae* Hoffmann cluster III, 15.6 %) and ST171 (* Enterobacter xiangfangensis *, 9.4 %); for *E. coli,* they were ST131 (29.2 %), ST10 (16.7 %) and ST127 (8.3 %). The phylogenetic relationship of the isolates for *E. coli *, species of the *E. cloacae* complex and *K. pneumonia* are shown in (Fig. 3).

Notably, we identified a new *K. pneumoniae* MLST (ST5000) closely related to ST11, which emerged in Cantabria (Fig. 3b). ST5000 is a single-locus variant possessing the mdh 113 allele instead of the mdh 1 allele as in ST11. This allele has a single-nucleotide substitution with respect to mdh allele 1 and, based on the even distribution of the approximately 35 single-nucleotide variants we identified between the main chromosomes of ST5000 and ST11 isolates, we conclude that the ST5000 has arisen as the result of random mutation as opposed to a recombination event or horizontal gene transfer. The negligible level of divergence between ST11 and ST5000 observed suggests that only a small amount of genetic drift has fortuitously affected one of the MLST loci and that they can be grouped together in a manner similar to how ST512 and ST258 are grouped together.

In contrast, we observed that ST1961 is closely related to the high-risk clone ST15 and actually shares phylogenetic classification based on the core genome phylogeny. However, distinguishing ST15 from ST1961 was the presence of OXA-48 on an IncL plasmid in ST15 rather than KPC on an IncP6 plasmid in ST1961. This can be seen more clearly in Fig. S13.

### Resistance gene annotation

After automatically annotating all protein-coding genes and identifying resistance genes in the final hybrid assemblies, we focused specifically on curating high-quality annotations of carbapenemase genes, the main genetic determinant of carbapenem-resistance in CPE isolates. The identification of carbapenemase genes was based on the consensus of three search methods and corresponding databases (Resfinder, AMRfinder and RGI). Conflicts between this consensus and the phenotypic classification performed at the hospital of origin were resolved using multiplex PCR for Enterobacterales carbapenemases and additional phenotypic assays [39].

Table 1 summarizes the results of this step. The breakdown by sequence type and chromosomal versus plasmid localization is shown in Fig. S13. It is readily apparent that the carbapenemase genes in the CPE isolates in this study are predominantly located in plasmids. The only appreciable accumulation of chromosomally located carbapenemase genes was that of OXA-48 genes (>20) in ST11. The only sequence type for which more carbapenemase genes were found in chromosomes than plasmids was ST127, also OXA-48 in this case.

**Table 1.** Results of the annotation of the main carbapenem resistance genes of interest

	OXA-48	KPC	VIM	NDM	IMP	GES
Original metadata (M)	315	95	46	12	2	1
Confirmed with gene prediction: M+/GP+	291	85	42	9	2	1
Confirmed with PCR: M+/GP?/PCR+	9	6	0	1	0	0
Contamination/conflict: GP≠PCR	3	2	2	1	0	0
Not confirmed: M+/GP−/PCR−	12	2	2	1	0	0
Metadata incorrect: M−/GP+/PCR+	3	0	1	0	2	0
**Final: A+B+E**	303	91	43	10	4	1

M, metadata; GP, gene prediction (gene found in the assembly); PCR, PCR on glycerol stock.

### Plasmids

Plasmid sequences were identified by size, circularity and similarity using PlasmidFinder and MOB-typer. In total we identified 1652 non-redundant plasmids, 1206 of them belonging to *K. pneumoniae* samples, of which 297 were found to contain carabapenemase genes. Typical size distributions were observed (Fig. S14): there are three main peaks at 40, 65 and 115 kb, respectively. This is consistent with previous studies [47], where IncF plasmids in Enterobacterales range from 45 to 200 kb. However, we identified homology to plasmid sequences in 11 scaffolds larger than 1 Mb, which could either represent assembly artefacts or actual plasmid integrations into the bacterial chromosome. Closer inspection of ONT read alignments at these loci uncovered four cases where the coverage dropped (e.g. AI2917_v1 and AI2912_v1 assemblies, Fig. S15a, b) and three cases where it doubled (e.g. AI2920_v1 assembly, Fig. S15c) at the boundaries of the putatively integrated plasmid sequence; we suspect that these were misassemblies. However, we also found four clear cases with even coverage in the region (e.g. the AI2705_v1 assembly, Fig. S15d), supporting integration of plasmid sequences.

**Fig. 4.**
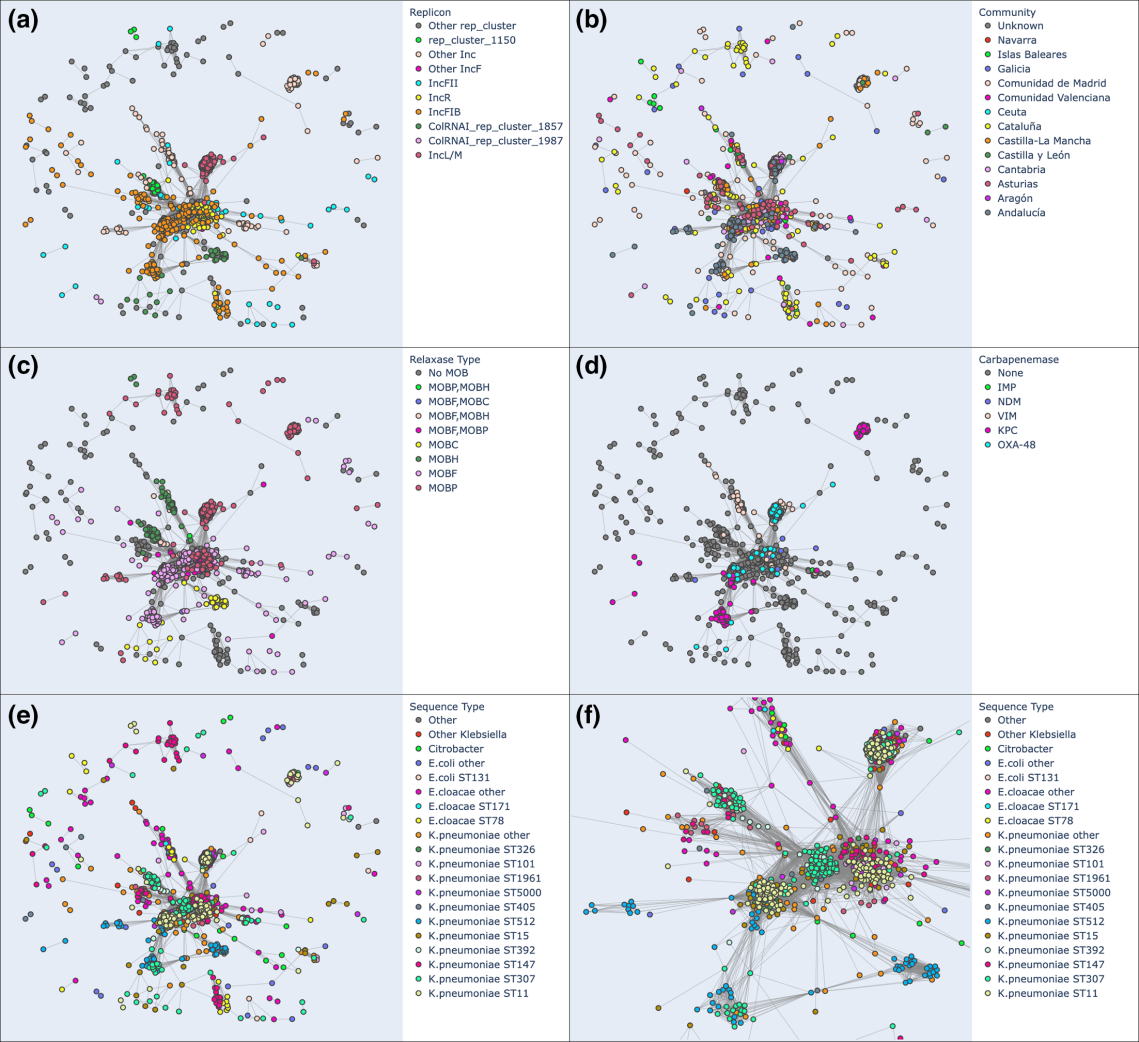
Clustering of plasmid sequences by average nucleotide identity. Plasmid sequences defined as sequences with length <1 Mbp identified by PlasmidFinder were clustered using the similarity metric tANI. Plasmid types (replicons and MOB types) are labelled in (a, c), the carbapenemase gene that they carry in (d), the community where they were isolated in (b), and the species and sequence type in (e, f) (zoomed in). The plasmid similarity network shown here can be explored interactively at https://resistome.cnag.cat/incredble/ani/, where these images were generated.

The remaining identified plasmids (sequences less than 1 Mbp in length identified by MOBTyper) were clustered based on similarity, using the total ANI (tANI) metric [43], which in turn is a function of ANI and AF. The resulting clusters are shown in Fig. 4. Focusing on different parts of this plasmid similarity network, we can make new observations that would be more difficult using the standard database search tools. For example, if one focuses on the KPC-containing MOBP plasmid cluster [panels c (red) and d (pink)] on the upper right side of the figure, which corresponds to the MASH cluster 891 (https://resistome.cnag.cat/incredble/scaffolds/?mash_neighbor_cluster=891), it is apparent that while this 40 kb plasmid is present in a diverse set of species (* E. coli *, *E. cloacae *, *C. freundii *, *Klebsiella, Raoultella ornithinolytica*) and strains, it is primarily present in only two communities, Galicia and in and around Madrid, with the plasmids found in Galicia mostly present in *K. pneumoniae* ST1961 (all isolates of ST1961 came from Pontevedra and carry this KPC-2-bearing plasmid), with a couple in *E. cloacae* complex ST515 and one in *R. ornithinolytica *, and those in Madrid present in a wider diversity of sequence types. Identifying these cases can be extremely useful for visualizing those plasmids that are strain-promiscuous and have spread to many locations. In another example, in panel f of the figure, one can see an OXA-48-containing IncL/MOBP cluster that is present in a wide variety of sequence types, while other clusters pertain to only to a few, for example, the KPC-containing plasmid cluster at the bottom of panel d found predominantly in ST307 and ST512 (panel f). In fact, the ST512 plasmids are predominantly present in Andalucia and nowhere else. One can also observe spread of the OXA-48 gene outside of the main IncL/MOBP cluster, but still falling within the same connected component. The VIM and NDM carbapenemases (mainly E. coli and *K. pneumoniae* ST101) can be seen to spread in a similar manner.

### Genes enriched in *K. pneumoniae* ST15

**Fig. 5.**
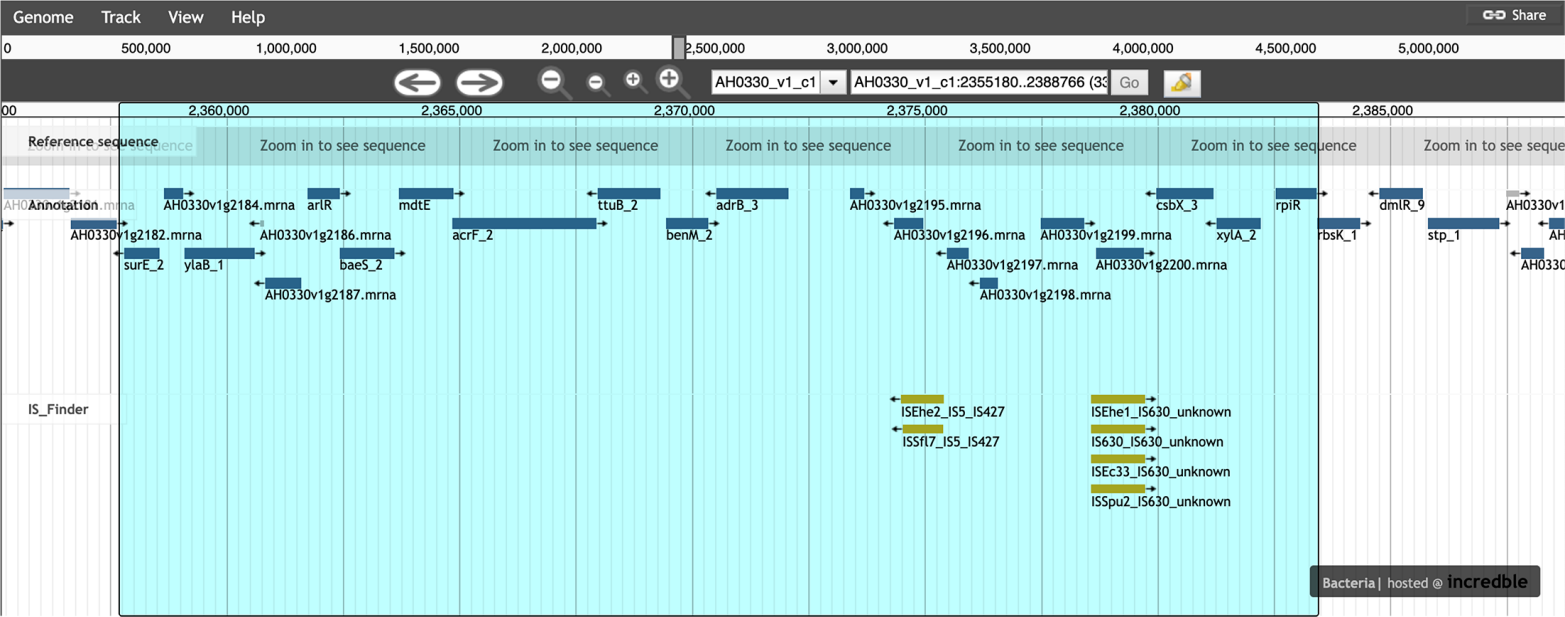
Representative ST15-unique gene cluster in the assembled genome of sample AH0330, an OXA-48-positive *K. pneumoniae* isolate collected at the Hospital Universitario Central de Asturias (Oviedo, Spain). Sample details at https://genomes.cnag.cat/incredble/277/.

Enrichment analysis revealed many genes present in the high-risk clone ST15 (and the highly related ST1961 and ST326) that are mostly absent from other sequence types. The top genes (present in >85 % of ST15 samples and <5 % of other STs) are listed in File S1. Some of these appear to have resistance- or virulence-related functions and a number of them are co-localized in a cluster of ~20 genes, an example of which is shown in Fig. 5. We suspect that AlrR (involved in the regulation of adhesion, autolysis, multidrug resistance and virulence in *S. aureus*) and mdtE and acrF (components of tripartite RND-type multidrug efflux pumps) may play an important role in ST15 spread. Interestingly, this gene cluster could not be found in its entirety in the short read-only SPAdes assembly generated by Unicycler before resolving the assembly graph with nanopore reads. Contiguity was lost at each of the interspersed repeats shown in the figure.

## Discussion

We have collected and sequenced 461 new CPE isolates from Spain using a combination of long and short reads, assembling them and annotating them with full clinical, phenotypic and genomic features. The hybrid assembly strategy used here has enabled us to obtain complete circular chromosomes and plasmids for over 83 % of isolates included in this study, which with Illumina-only approaches is not possible. This is in contrast to some recent large-scale projects aimed at sequencing carbapenemase-producing Enterobacterales that have opted for assembly [5] or merely resequencing and mapping [48] of short read-only (next-generation sequencing) sequencing data. A prerequisite for identification of transmission clusters or events is the accurate identification and characterization of resistance- or virulence-transmitting plasmids, which often harbour carbapenemases. Only long-read or hybrid sequencing and assembly methods are able to consistently provide this ability. Proof of principle that nanopore sequencing can achieve these goals has previously been demonstrated [11, 49, 50], albeit on a limited numbers of samples. Furthermore, the development of informatics pipelines such as pathoLogic tailored to plasmid identification can play an instrumental role in genomic surveillance of AMR [50]. Our work is differentiated from these studies in that we combine the long-read sequencing of the latter studies with the scale of the former studies, and achieve a high circularization rate. While our AACRE pipeline is similar to the pathoLogic pipeline, we have automated more steps, included species quality control and additional analysis steps.

By sequencing and assembling a substantial number of samples at high depth and with different ONT chemistries, we concluded that the ONT coverage needed to obtain circular bacterial assemblies should be no lower than 30× and ideally more than 80×, with early R10 chemistry requiring higher coverage to achieve similar circularity ratios. We showed that both ONT/Illumina hybrid assembly and ONT-only assembly using R10 chemistry result in similar assembly and annotation quality, while Illumina-only assembly results in more fragmented and incomplete assemblies and annotations. With further increases in accuracy for ONT nanopore sequencing chemistry (R10.4 and kit 14) and base calling (CRF-based super accuracy mode, duplex reads), we foresee the simplification of bacterial genome sequencing (single library preparation) while maintaining assembly and annotation accuracy. Indeed, recent studies [51, 52] performed with the same versions of the chemistry used in the present study confirm our results.

Finally, we made it a goal to organize the genomic, clinical and microbiological data in an easily searchable relational database to further enhance the value of the data collected. As a complement to repository submission and subsequent propagation to already-established databases, we wanted something fast and lightweight that could facilitate the query, comparison and display of genomic results. While other already available AMR databases are quite powerful and comprehensive, we found that they only partially met these criteria. The NCBI’s National Database of Antibiotic Resistant Organisms, for example, has an Isolates Browser [53] that, while comprehensive, is difficult to navigate. The data available via PATRIC (University of Chicago) [54] are extensive, including filters, detailed views and genome browsing, but for certain regions such as Spain the number of complete Enterobacterales genomes available has been limited. Finally, the Microreact site that hosts the EuSCAPE *K. pneumoniae* data [55] provides useful map and tree views but only basic filtering tools and again is limited in terms of Spanish isolates. Therefore, we developed inCREDBle, a database and web front end for rapid exploration of long read-based complete genomes of clinical isolates of carbapenem-resistant carbapenemase-producing Enterobacterales from Spain. The current contents of inCREDBle constitute a regional snapshot of carbapenem resistance, which, combined with the currently implemented genomics-oriented search and comparison features, represents a new resource for AMR surveillance.

Storage in inCREDBle of comparative data, such as Roary groups and orthogroups calculated for genes, as well as MASH neighbour clusters and plasmid identification features at the sequence level, has enabled much more rapid exploration of the data set we produced. For example, when we hypothesized that the presence or absence of particular genes might contribute to the spread of ST15 in the population, we decided to use the database to search for presence/absence variation specific to ST15, first by browsing accessory genes in ST15 using the web interface (requiring no programming knowledge) and then by using a simple Python script to carry out the queries more precisely and with *P*-values for enrichment. This analysis revealed a cluster of genes present in ST15 (and the highly related ST1961 and ST326) that are mostly absent from other sequence types. Some of these appear to have resistance- or virulence-related functions. Of note, short read-only methods failed to assemble this gene cluster intact. Additional comparative data, for example sequence similarity metrics such as tANI that we calculated for all sequences added to inCREDBle, can be stored as well, enabling the dynamic generation of new or updated interactive similarity networks on the server when adding new samples or the serving of cached versions between database updates. We note that such comparative features are similar in nature to the Ensembl compara database, thus necessitating versioning of the database on a periodic basis, which would require continued technical support.

Looking to the future, we would like to apply our ONT-based genome sequencing and AACRE–inCREDBle bioinformatics solution to the task of genomic AMR surveillance. The whole workflow is modular in nature, which makes it adaptable to different situations, including integration into clinical microbiology services. Culturing of isolates and DNA extraction can be performed in a distributed manner, if necessary, with genome sequencing and assembly either distributed or centralized thanks to the portability and affordability of the ONT platform and the flexibility of the AACRE Snakemake pipeline. The annotation, analysis and database entry tasks, though, can easily be done centrally, via a web service, to ensure uniformity in processing and the ability to carry out comparative analyses such as Roary core/accessory genome construction, phylogenetic inferences and enrichment analyses on a periodic basis. With the routine unbiased addition of more strains, both resistant and susceptible, to the inCREDBle database, and the development and addition of tracking features to inCREDBle, we aim to enhance regional and national AMR monitoring capabilities.

## Supplementary Data

Supplementary material 1

Supplementary material 2
