# Supplementary material for: Development of a novel streamlined workflow (AACRE) and database (inCREDBle) for genomic analysis of carbapenem-resistant Enterobacterales
Source: Microb Genom. 2023 Nov 27;9(11):001132. doi: 10.1099/mgen.0.001132 (PMC10711315; doi:10.1099/mgen.0.001132)
Supplement: Supplementary material 1 [file mgen-9-1132-s001.pdf]

SUPPLEMENTARY INFORMATION

FIGURES

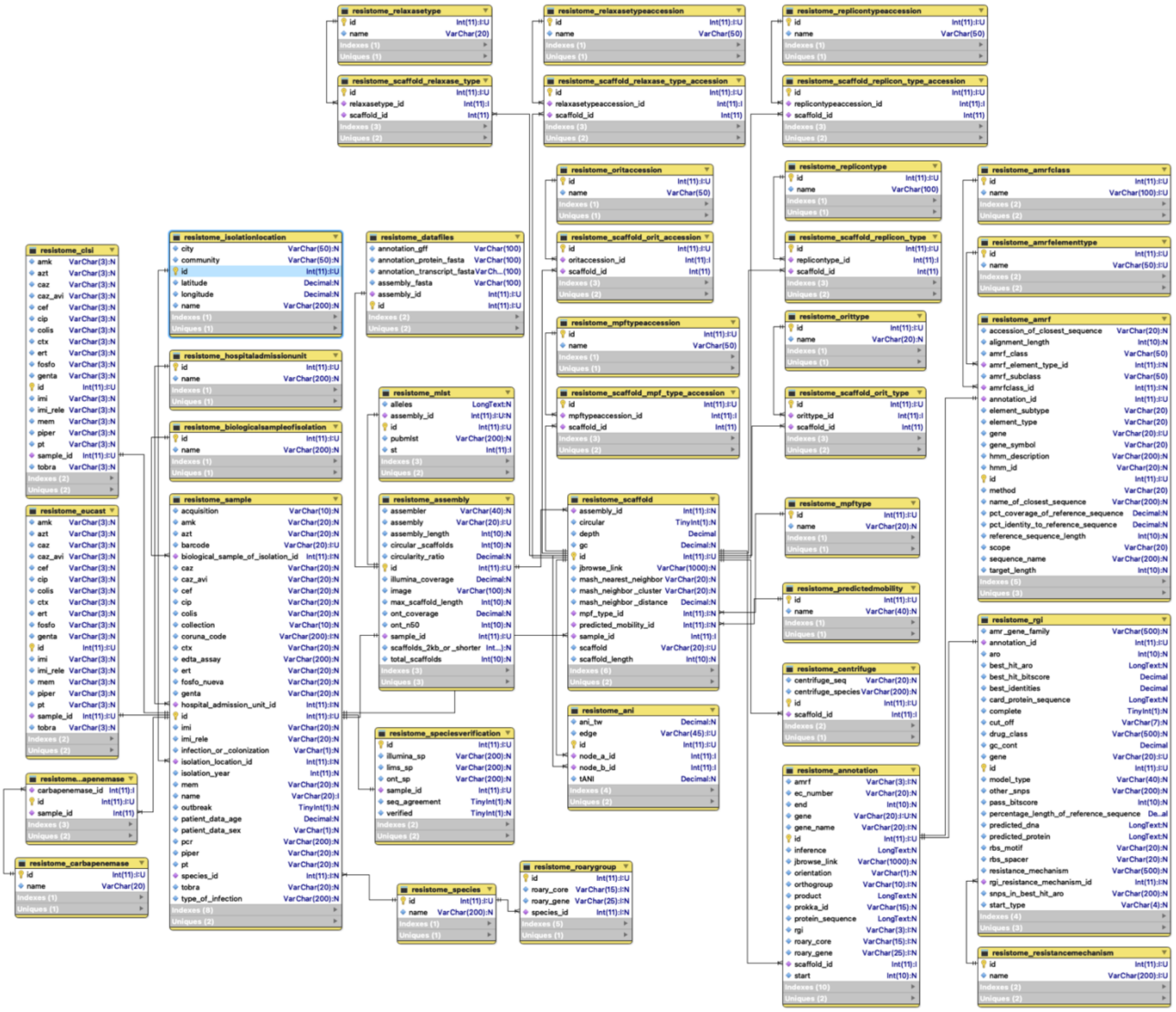

Figure S1. The inCREDBle database schema.

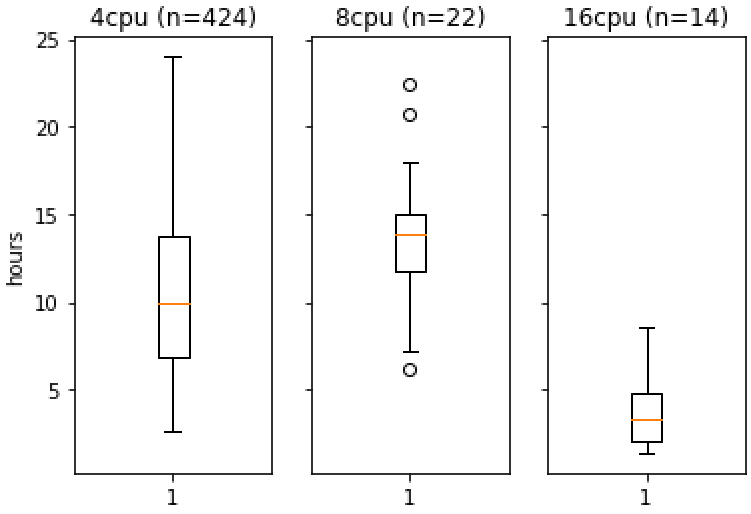

Figure S2. AACRE pipeline performance. The distribution of running times by number of threads used for assembly of each sample. is shown.

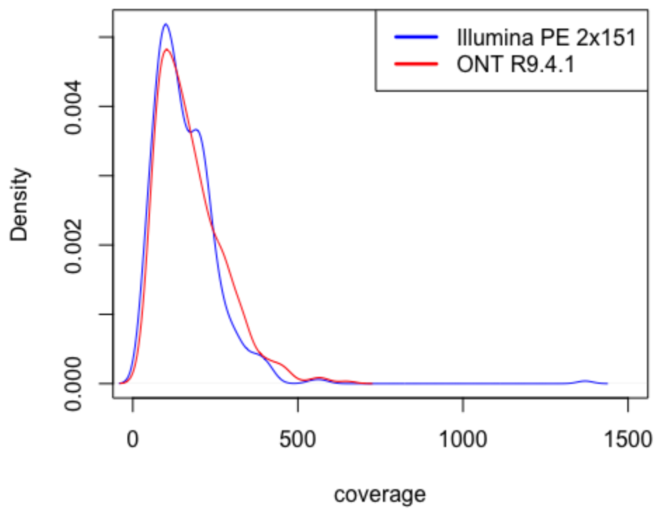

Figure S3. The distributions of Illumina versus ONT R9 genome coverage.

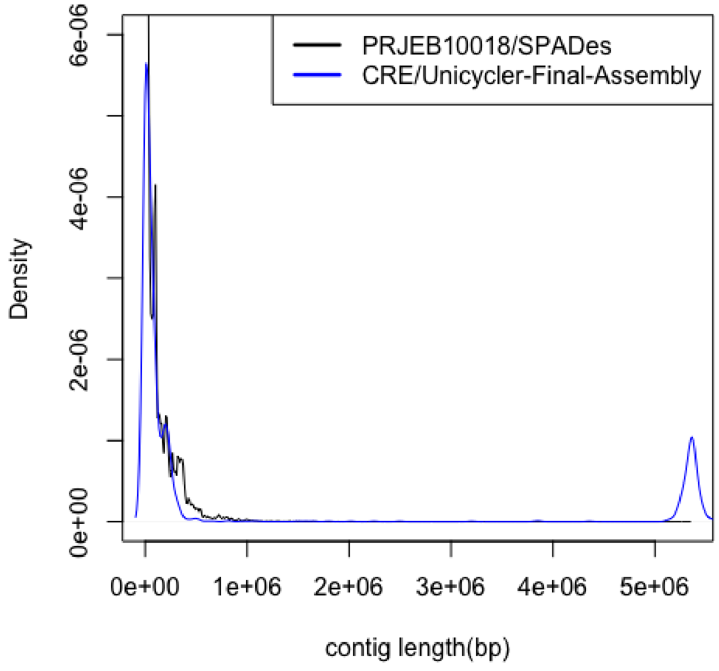

Figure S4. Distribution of scaffold lengths of SPAdes short-read assemblies and Unicycler hybrid assemblies. The comparison was done using scaffolds longer than 292 bp to discard the chaff from both assembly sets (CRE, this paper, and PRJEB10018).

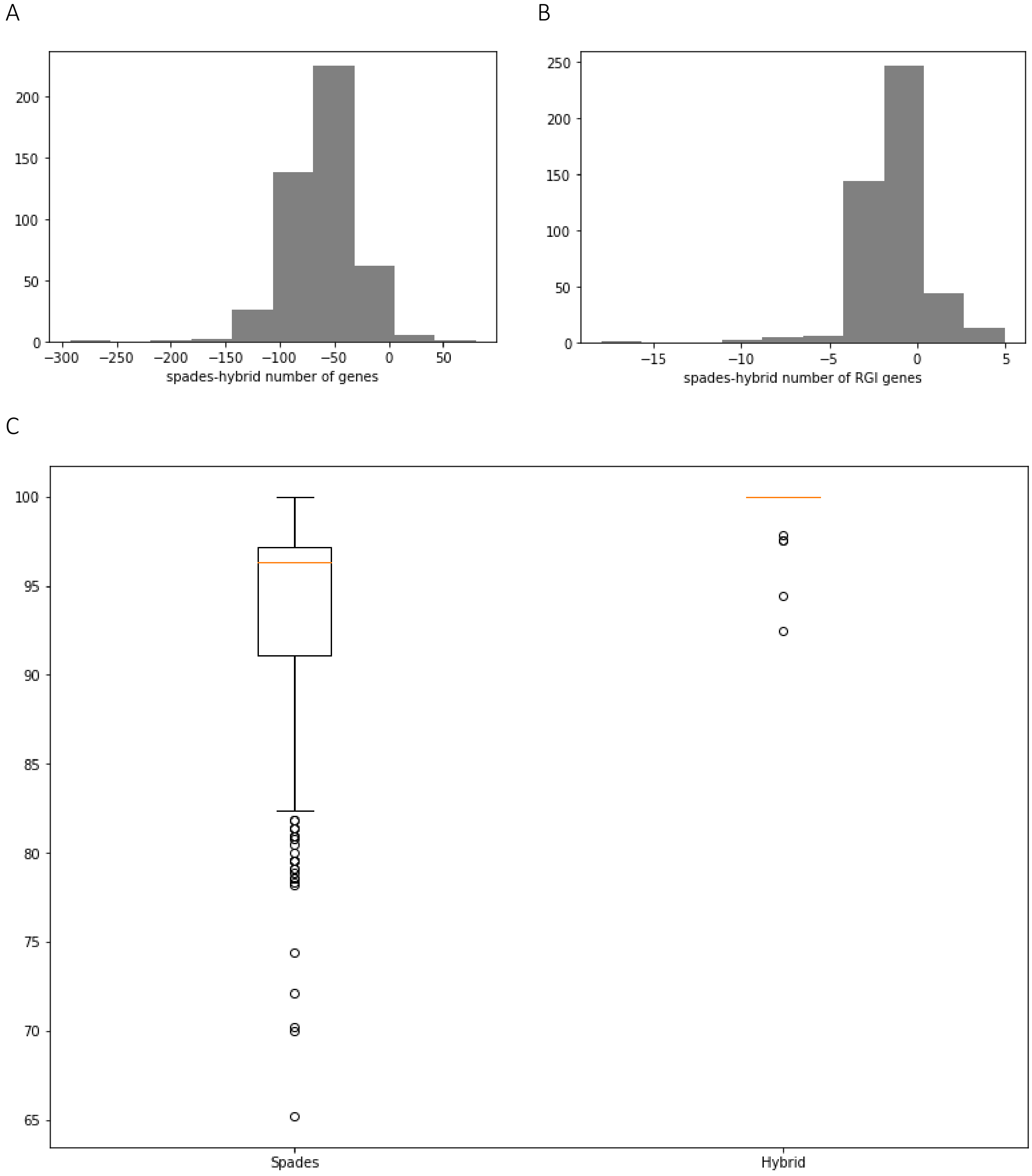

Figure S5. Annotation comparison between Unycicler hybrid and SPADes Illumina assemblies. Histograms show the difference in number of annotated genes between the Illumina-only and hybrid assemblies of each sample (A) and the differences in the number of annotated resistant genes (RGI) between the Illumina-only and Hybrid assemblies of each sample (B). Boxplots of the RGI gene completeness ratios for Illumina-only and Hybrid assemblies are shown in (C).

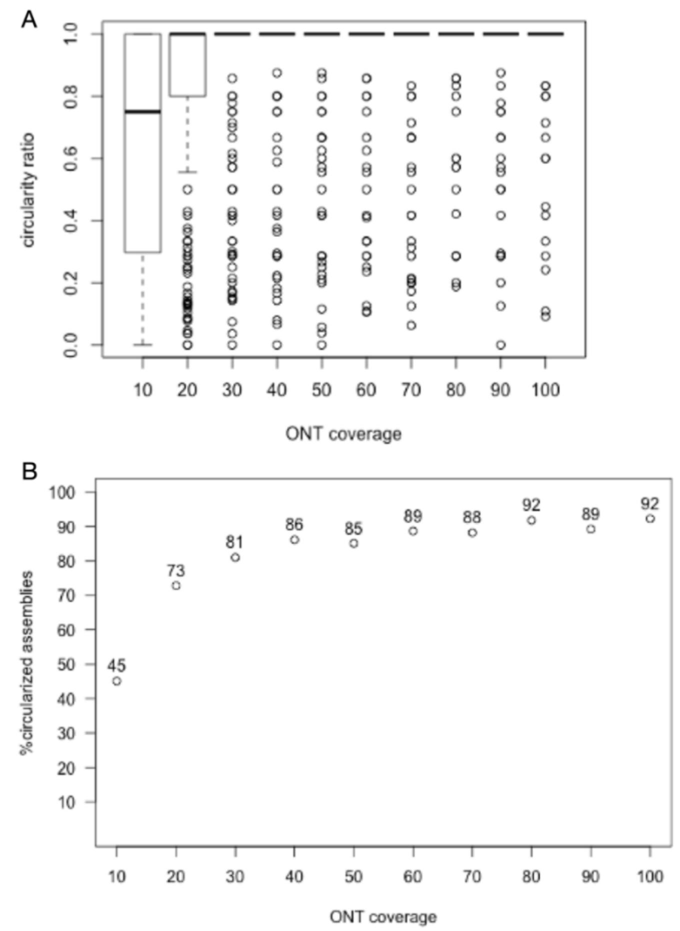

Figure S6. Effect of lower ONT coverage for well-assembled samples at high coverage. We downsampled the ONT read data for 195 well-assembled samples (circularity ratio = 1) to 10x - 100x in 10x increments maintaining the original Illumina coverage, and then assembled them in bold mold with the AACRE pipeline. Here we show (A) the circularity ratio versus ONT coverage obtained with R9 (Spearman's rank correlation: rho = 0.282, p-value < 2.2e-16) and (B) the percentage of fully circularized assemblies for each R9 coverage slice (Spearman's rank correlation: rho = 0.9636364, p-value < 2.2e-16).

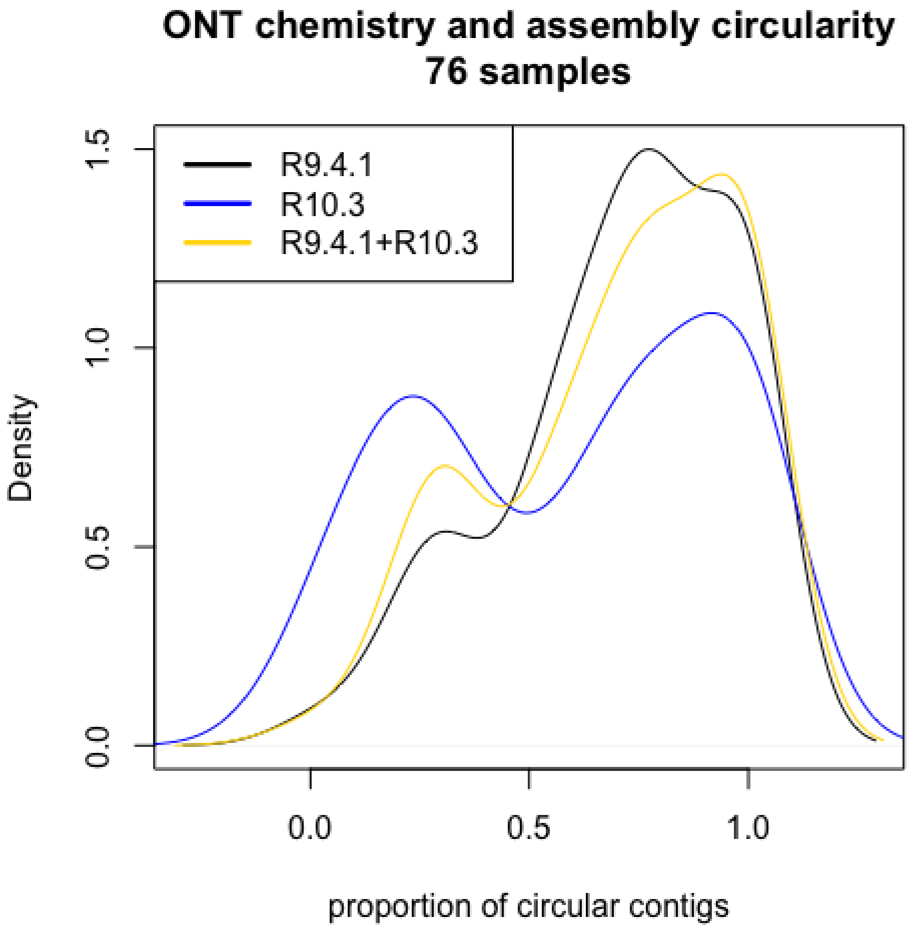

Figure S7. Circularity ratio distribution by ONT chemistry. These 76 samples had been initially assembled and annotated with the AACRE pipeline using the ONT reads obtained with the R9.4.1 chemistry. We then ran the AACRE pipeline combining the Illumina reads with either just R10.3 read data or both R9.4.1 and R10.3 data.

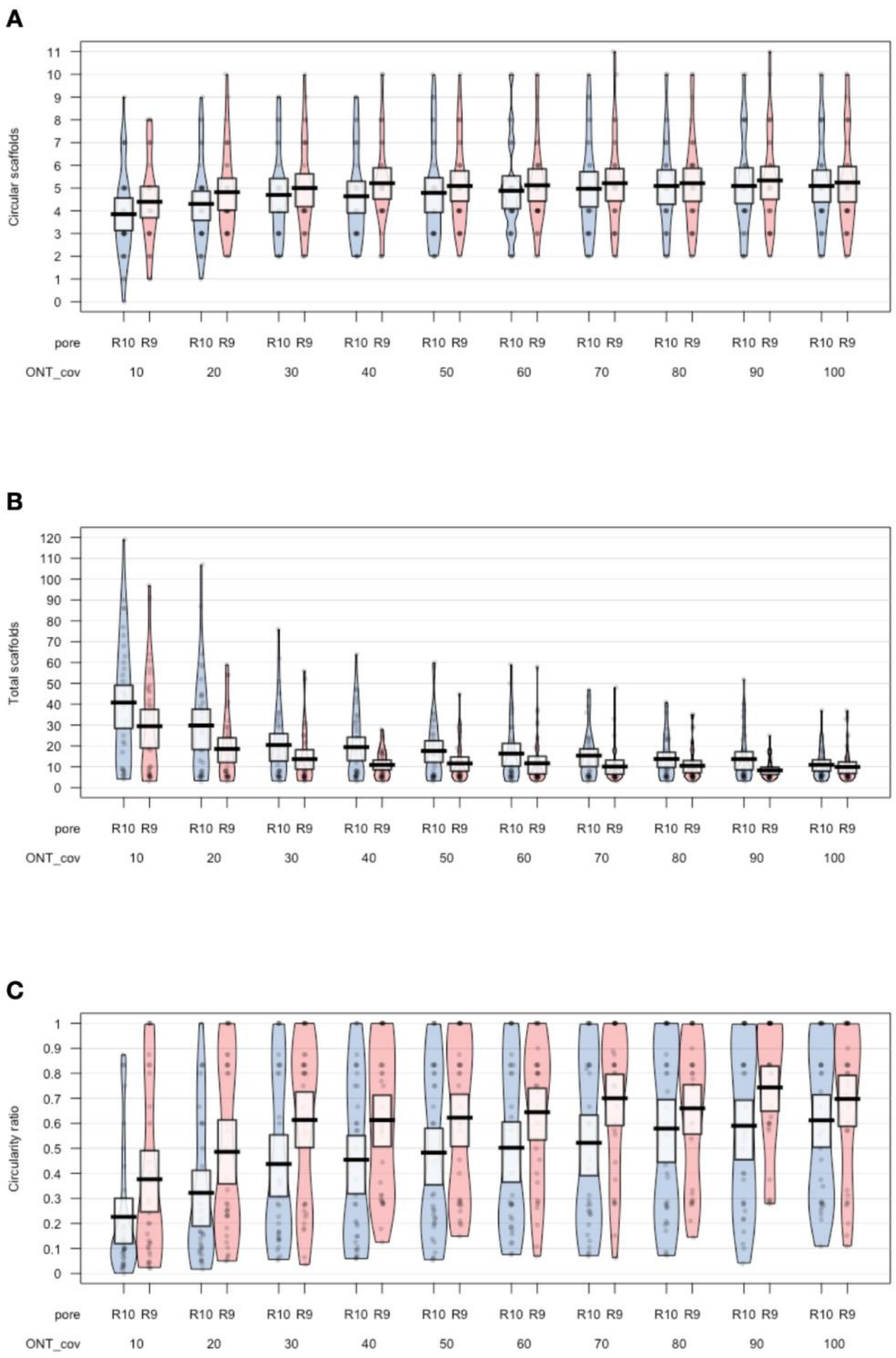

Figure S8. ONT scaffold circularization by ONT coverage. To compare the effect of coverage between the two chemistries on hybrid assembly, we downsampled both the R9 and R10 read data to equivalent coverages from 100x down to 10x. Of the 76 samples, 33 had at least 100x coverage of both R9 and R10. Note that this dataset contained samples that were more problematic and difficult to assemble. Shown are the number of circular scaffolds (A), the total number of scaffolds (B), and the circulariity ratio (C) versus ONT coverage obtained with both R10 and R9 (N=33). Median values (dark bars) and quartiles (white boxes) are shown.

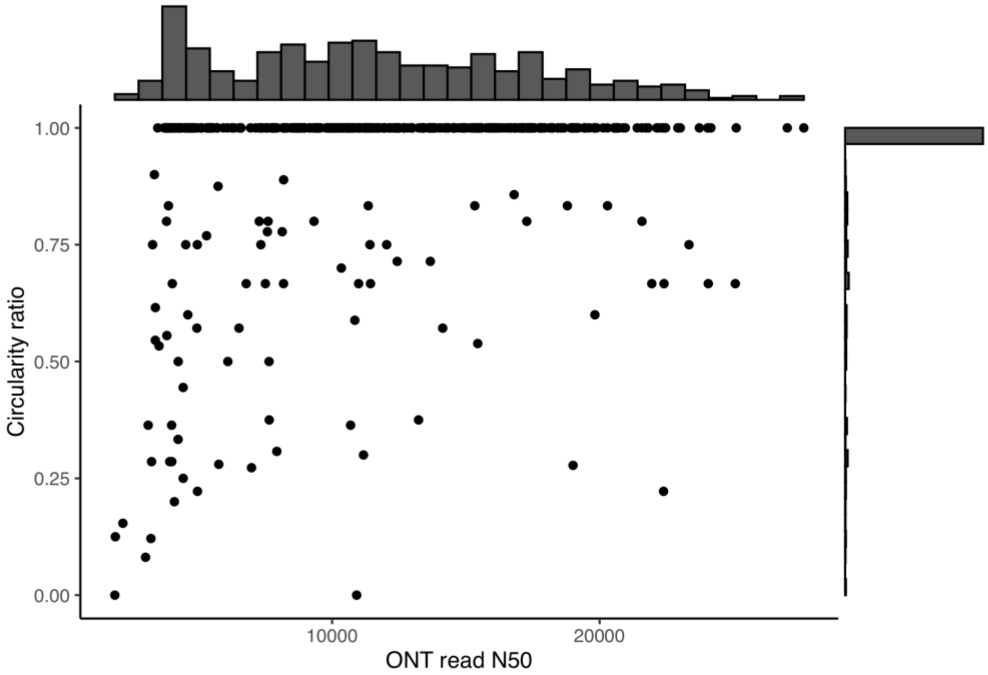

Figure S9. ONT R9 read N50 versus circularity ratio. Marginal densities are shown as histograms. N=461.

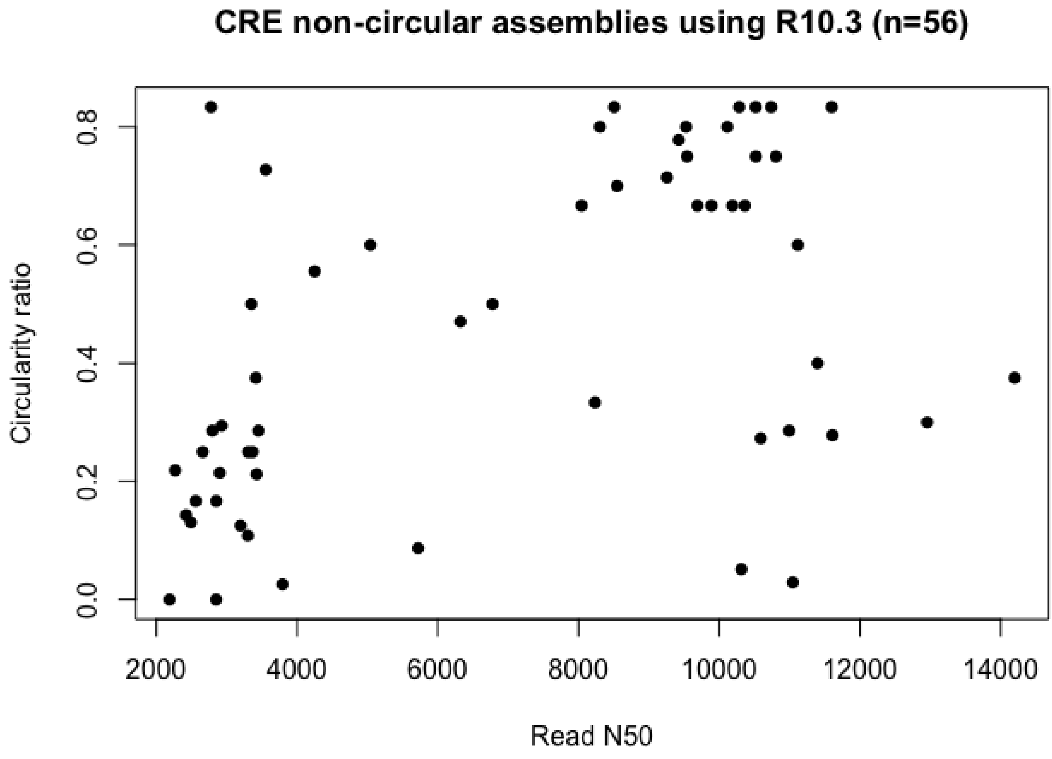

Figure S10. ONT R10 read N50 versus circularity ratio. The Spearman's Rank Correlation rho = 0.47 (p-value = 0.00026). N=56.

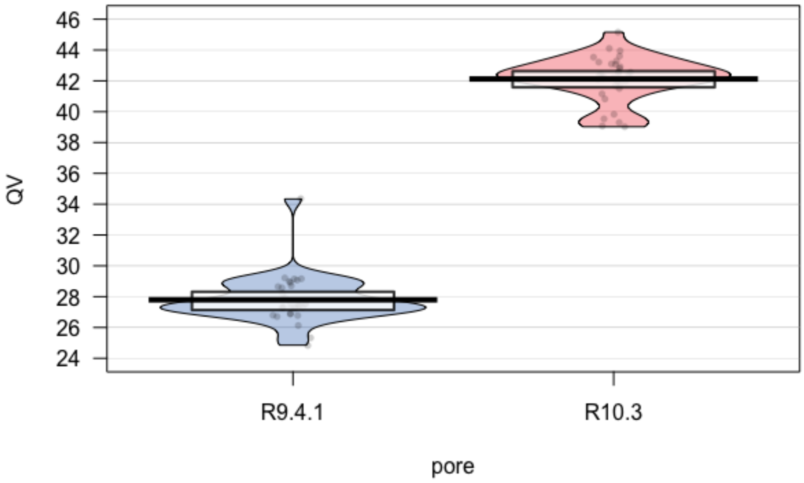

Figure S11. Comparison of consensus quality for different ONT Chemistries. Shown are QV value distributions determined by Mummer alignment of long-read only assemblies (Flye plus Racon and Medaka polishing) of either ONT R9 reads or R10 reads to the Unicycler hybrid assembly (R9 plus Illumina paired end reads). Error rates were calculated as the total number of single nucleotide and indel differences divided by the number of aligned bases in 1-to-1 alignments as reported by *dnadiff*. The error rates were converted to QV values using the Phred scale. QV40 = error rate of 1 in 10,000. QV30 = 1/1000.

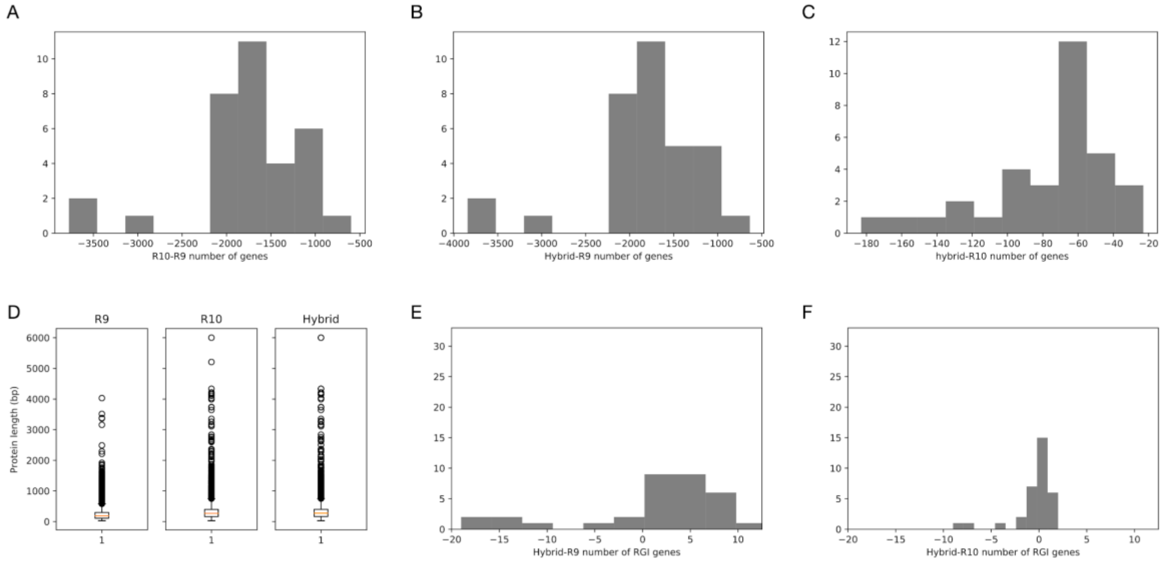

Figure S12. Comparison of gene annotations in long-read assemblies and hybrid assemblies. (A) The distribution of differences in annotated gene number between the R10 and R9 long-read Flye assemblies (33 samples each). A negative value on the x-axis indicates that the R9 assembly had more genes annotated than the R10 assembly of the same sample. (B) The same but comparing the number of genes in hybrid assemblies versus their R9 long-read counterpart. (C) Hybrid versus R10. (D) Boxplots showing the difference in gene lengths among R9, R10 and hybrid assemblies. (E) The difference in the number of RGI gene annotations between hybrid and R9 assemblies. Note that a positive value indicates fewer RGI genes were found in the R9 Flye assembly. (F) The difference in the number of RGI gene annotations between hybrid and R10 assemblies.

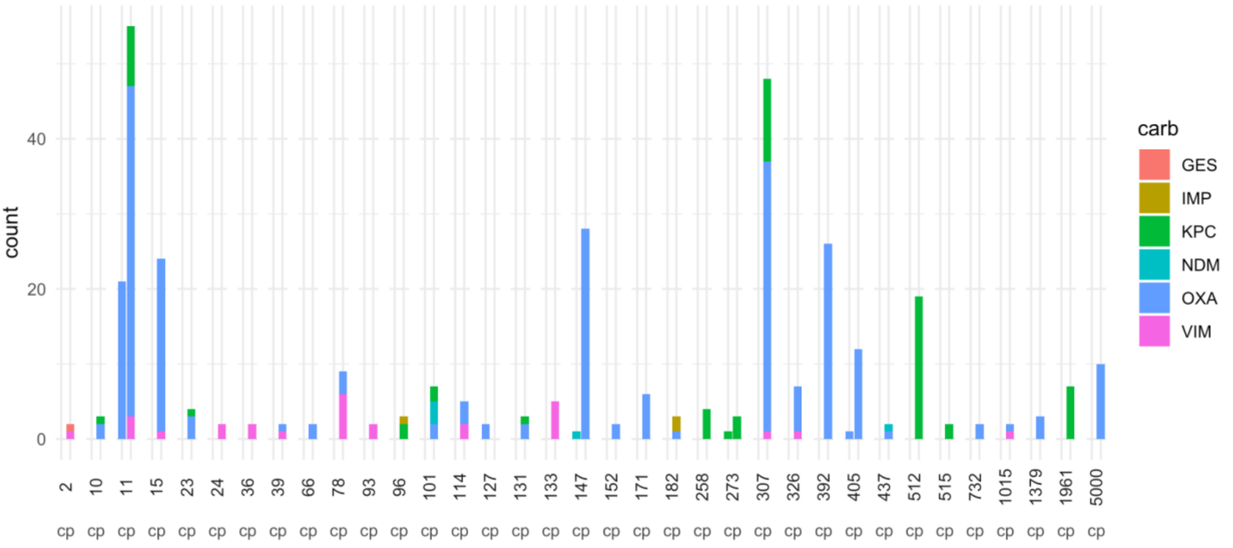

Figure S13. Carbapenemases by Sequence Type. The cumulative count of carbapenemases per sequence type (x-axis) is shown for both main chromosomes ("c") and plasmids ("p). The stacked bars are colored according to carbapenemase type: GES=GES-6; IMP=IMP-8,IMP-13; KPC=KPC-2,KPC-3; NDM=NDM-1,NDM-23; OXA=OXA-48; VIM=VIM-1.

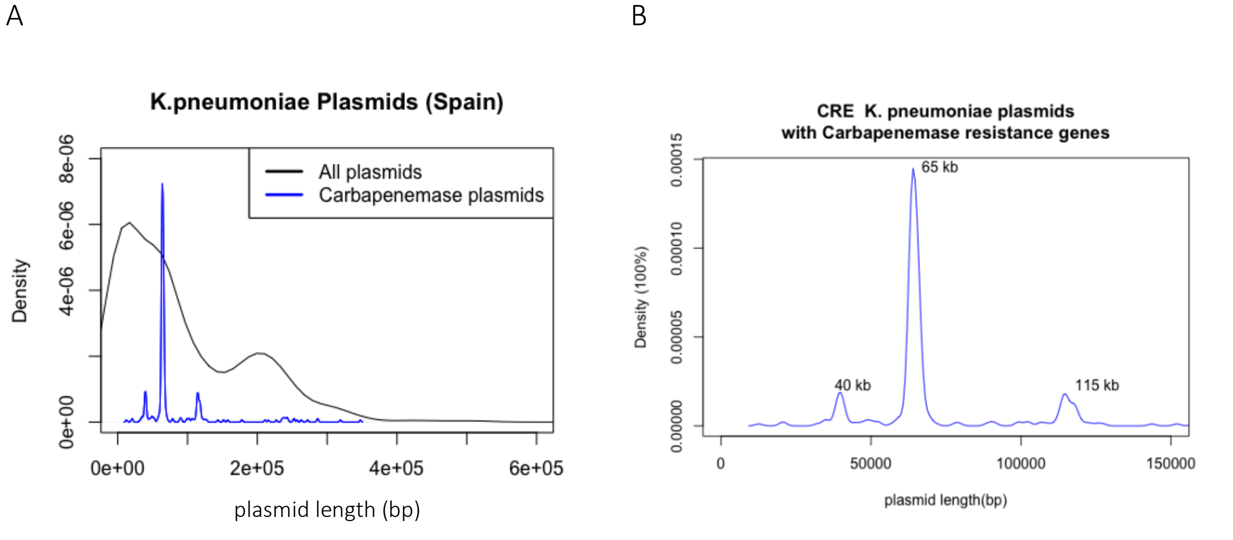

Figure S14. Sequence length distribution of identified plasmids. A) Distribution of the lengths of scaffolds corresponding to plasmids in the *K. pneumoniae* samples. The distribution for all plasmids is shown in black, while the distribution for those containing carbapenemase genes is shown in blue. B) A closer view of the lengths of carbapenemase-containing plasmids is shown.

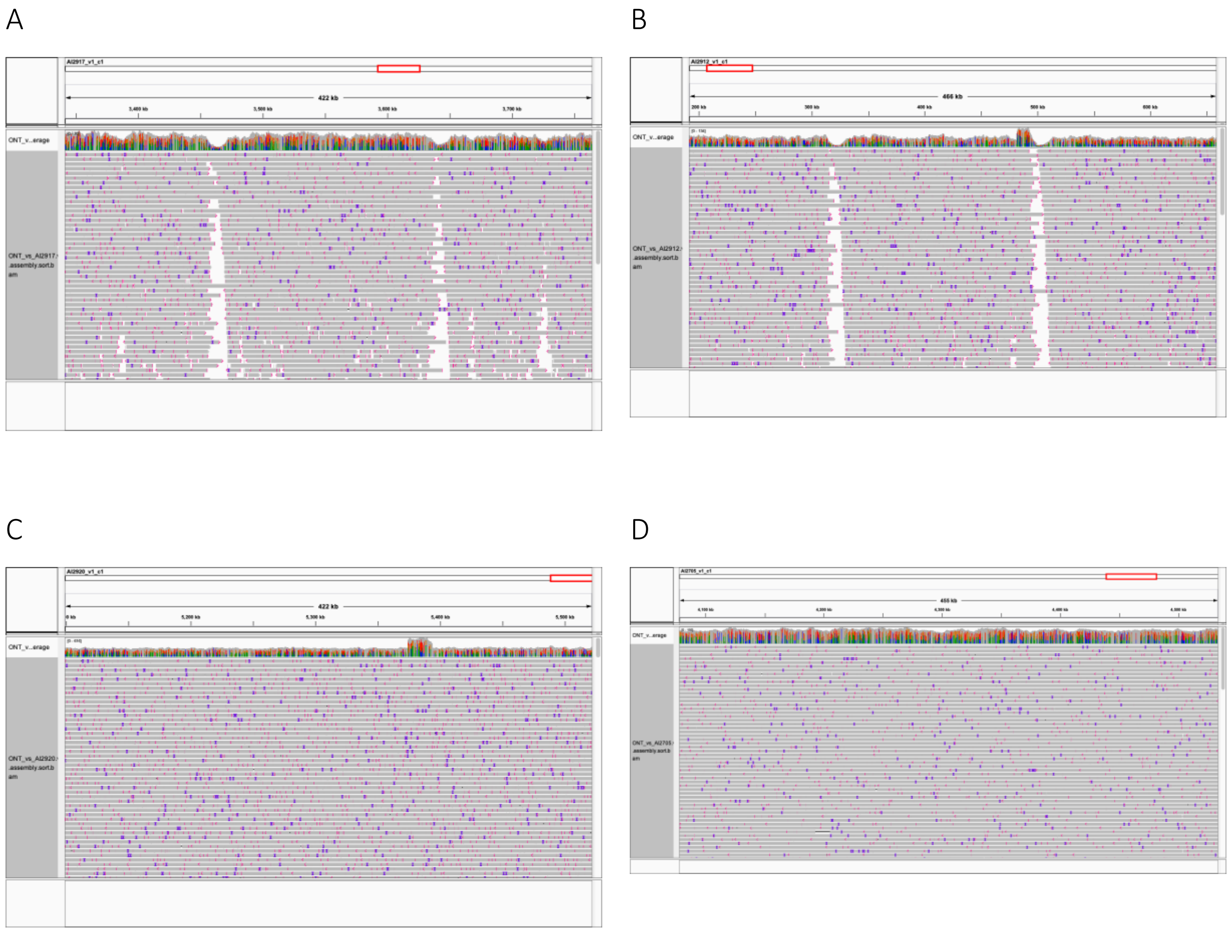

Figure S15. Read alignments at putative plasmid insertion sites. Four example IGV snapshots are shown: a coverage drop around putative plasmid insertion in AI2917_v1_c1 (a), a coverage drop around putative plasmid insertion in AI2912_v1_c1 (b), a repeat-induced coverage increase around putative plasmid insertion in AI2920_v1_c1 (c), and even coverage around putative plasmid insertion in AI2705_v1_c1 (d).

TABLES

Table S1. Assembly and Sequencing Statistics. The median assembly length of 5,640,631 bp was used to standardize the coverage estimate across samples regardless of the assembly efficiency.

Metric	Min.	1st Qu.	Median	Mean	3rd Qu.	Max.	SD
Assembly Length (bp)	4,741,709	5,461,519	5,640,631	5,584,959	5,744,640	6,468,007	284,594
Maximum Scaffold Length	462,118	5,184,627	5,334,698	5,190,386	5,378,130	6,183,346	497,395
N50	203,558	5,184,627	5,334,698	5,182,856	5,378,130	6,183,346	548,127
No. scaffolds	1	4	5	6.53	7	325	15.6
No. circular scaffolds	0	3	5	4.86	6	12	1.95
Circularity Ratio	0	0	0	0.93	0	0	0.19
No. Scaffolds ≤ 2 kb	0	0	0	1.2	0	241	11.6
Illumina sequencing coverage	26.6	89.7	138.6	159.2	206.9	1,369.8	103.4
ONT sequencing coverage	38.3	96.5	153.3	175.8	228.4	647.8	99.4

Table S2. Summary of scaffold lengths per assembly set.

	Min.	1st Qu.	Median	Mean	3rd Qu.	Max.	SD
Unicycler-hybrid (this study)	104	4164	63,589	963,608	214,043	5,663,976	1,971,505
SPAdes-Illumina (this study)	89	149	388	20,826	3861	876,053	61,161
SPADes-Illumina (PRJEB10018)	200	294	775	37,907	10,716	5,349,525	110,356

Table S3. Assembly and annotation metrics of SPAdes Illumina assemblies versus Unicyclyer hybrid assemblies for the 461 samples included in this study. Mean and standard deviation are shown for each metric.

	Illumina assemblies	Hybrid assemblies
Assembly N50	180.57 ± 64.49 kb	5,190.38 ± 497.39 kb
Number of scaffolds	264.43 ± 73.79	6.53 ± 15.59
Number of circular scaffolds	0	4.86 ± 1.95
Longest scaffold length	501.02 ± 145.55 kb	5,190.38 ± 497.39 kb
Number of genes	5173 ± 263	5234 ± 271
Number of RGI genes	37 ± 8.51	38 ± 8.50
Complete RGI genes	35 ± 7.95	38 ± 8.50
Percent of genome annotated as IS	0.51%	1.08%

Table S4. List of plasmids identified inside bacterial chromosomes.

Sequence	Assembly Span	Rep Type(s)	Relaxase Type(s)	MASH Cluster	Read Coverage at Junction
AH0328_v1_c1	4975330	IncH, rep_cluster_1088	MOBH	429	decreased
AI2719_v1_c1	4978857	IncFIB, IncFII	MOBF	708	even
AI2705_v1_c1	5017737	IncFIB, IncFII	MOBF	708	even
AI2822_v1_c1	5517947	IncFIB, IncFII	MOBF	770	increased
AI2862_v1_c1	5530992	IncFIB, IncFII	MOBF	770	decreased
AI2920_v1_c1	5567936	IncFIB, IncFII	MOBF	770	increased
AI2912_v1_c1	5594246	IncFIB, IncFII	MOBF	770	decreased
AI2927_v1_c1	5663976	IncFIB, IncFII	MOBF	770	increased
AI2917_v1_c1	5590497	IncFII, IncFIB	MOBF	770	decreased
AI2618_v1_c1	5429141	IncP	MOBP	3040	even
AI2617_v1_c1	5480730	IncP	MOBP	3040	even

Table S5. Species diversity of 71 samples for which both ONT R9 and R10 sequencing was performed.

Species	Total samples
Klebsiella pneumoniae	58
Escherichia coli	6
Enterobacter cloacae	6
Citrobacter freundii	4
Klebsiella oxytoca	2
